# Clinical Characteristics, Symptoms, and Long-Term Outcomes in Gitelman Syndrome

**DOI:** 10.1016/j.ekir.2025.09.006

**Published:** 2025-09-04

**Authors:** Michiel L.A.J. Wieërs, Lise Allard, Viola D’Ambrosio, Pedro Arango-Sancho, Jeroen H.F. de Baaij, Francesca Becherucci, Aurelia Bertholet-Thomas, Martine Besouw, Anne Blanchard, Martina Cacciapuoti, Vincenza Carbone, Elisabeth A. Cornelissen, Federico Daffara, Jan Degenhardt, Olivier Devuyst, Eiske Dorresteijn, Rhys Evans, Lucile Figueres, Marc Fila, Marica Giliberti, Valentine Gillion, Sophie Haumann, Gerlineke Hawkins – van der Cingel, Pascal Houillier, Marguerite Hureaux, Felix Knauf, Bertrand Knebelmann, Martin Konrad, Theresa Kwon, Sandrine Lemoine, Germana Longo, Tom Nijenhuis, Rik H.G. Olde Engberink, Hector Ríos Duro, Chloé Saadé, John A. Sayer, Karl-Peter Schlingmann, Thomas Simon, Marijn M. Speeckaert, Hai Liang Tan, Francesco Trepiccione, Rosa Vargas-Poussou, Faidra Veligratti, Stephen B. Walsh, Mahdi Salih, Pedro H. Imenez Silva, Ewout J. Hoorn, Gitelman Survey Study, Pilar Auñón, Detlef Bockenhauer, Peter J. Conlon, Cosima Erhardt, Sacha Flammier, Cristina Blázquez Gómez, Sercin Guven, Gerlineke Hawkins, Jan Halbritter, Maria Herthelius, Alba Herreros, Dragan Klaric, Nóra Ledó, Pierluigi Marzuillo, Susanne Schäfer, Roland Schmitt, Malgorzata Stanczyk, Francesa Taroni, Nikola Zagorec

**Affiliations:** 1 Division of Nephrology and Transplantation, Department of Internal Medicine, Erasmus Medical Center, University Medical Center Rotterdam, Rotterdam, The Netherlands; 2 Pediatric Nephrology Unit, Centre de Référence Maladies Rénales Rares Sorare, Bordeaux University Hospital, Bordeaux, France; 3 UOC Nefrologia, Dialisi e Trapianto, Fondazione Policlinico Universitario “A. Gemelli” IRCCS, Rome, Italy; 4 Department of Pediatric Nephrology, Hospital Sant Joan de Déu, Barcelona, Spain; 5 Department of Medical BioSciences, Radboud University Medical Center, Nijmegen, The Netherlands; 6 Nephrology and Dialysis Unit, Meyer Children's Hospital IRCCS, Florence, Italy; 7 Department of Biomedical, Experimental and Clinical Sciences “Mario Serio,” University of Florence, Florence, Italy; 8 Pediatric Nephrology Unit, Hospices Civils de Lyon, Centre de référence Maladies Rénales Rares MAREGE – Filière ORKiD, Lyon, France; 9 Department of Pediatric Nephrology, Beatrix Children's Hospital, University Medical Center Groningen, University of Groningen, Groningen, the Netherlands; 10 Paris Cité University, Paris, France; 11 Reference Center of Rare Renal Disease of Children and Adults (MARHEA), Paris, France; 12 INSERM, Clinical Investigation Center CIC1418, Assistance Publique-Hopitaux de Paris, European Georges Pompidou hospital, Paris, France; 13 INSERM, UMRS 1138, Centre de Recherche des Cordeliers, Paris, France; 14 Nephrology, Dialysis and Transplantation Unit, Department of Medicine, University of Padova, Padova, Italy; 15 Department of Biomedical, Experimental and Clinical Sciences “Mario Serio,” University of Florence, Florence, Italy; 16 Department of Pediatric Nephrology, Amalia Children’s Hospital, Radboud University Medical Center, Nijmegen, The Netherlands; 17 Nephrology Unit, Department of Medical and Surgical Specialties, Radiological Sciences and Public Health, Spedali Civili Hospital, University of Brescia, Brescia, BS, Italy; 18 Department II of Internal Medicine, University of Cologne, Koln, Nordrhein-Westfalen, Germany; 19 Institute of Physiology, University of Zürich, Zürich, Switzerland; 20 Division of Nephrology, Cliniques universitaires Saint-Luc, UCLouvain Medical School, Brussels, Belgium; 21 Department of Pediatric Nephrology, Sophia Children’s Hospital, Erasmus Medical Center, Rotterdam, The Netherlands; 22 Department of Renal Medicine, London Tubular Centre, University College London Medical School, London, UK; 23 Nephrology, Nantes University Hospital, CR2TI ITUN, Nantes, UK; 24 Reference Centers for Rare Diseases of Calcium and Phosphate (Filière OSCAR) and Nephrogenetic (Filière ORKID), Paris, France; 25 Pediatric Nephrology Unit, Centre Hospitalier Universitaire Arnaud de Villeneuve-Université de Montpellier, Centre de Référence Maladies Rénales Rares Sorare, France; 26 Department of Precision and Regenerative Medicine and Ionian Area-Nephrology, Dialysis and Transplantation Unit, University of Bari “Aldo Moro”, Bari, Italy; 27 Department of Pediatrics, Pediatric Nephrology, Faculty of Medicine, University Hospital Cologne, University of Cologne, Cologne, Germany; 28 Department of Nephrology and Medical Intensive Care, Charité-Universitätsmedizin Berlin, Berlin, Germany; 29 Centre de Recherche des Cordeliers, INSERM, Sorbonne Université, Université Paris Cité, Paris, France; 30 Service de Médecine Génomique des Maladies Rares, Hôpital Européen Georges Pompidou, Groupe Hospitalier Universitaire Centre, Assistance Publique Hôpitaux de Paris, Paris, France; 31 INSERM, PARCC U970, F-75015 Paris, France; 32 Division of Nephrology and Hypertension, Department of Internal Medicine, Mayo Clinic, Rochester, Minnesota, USA; 33 APHP, Service de Nephrologie, Hôpital Universitaire Necker, Université de Paris, Paris, France; 34 Department of General Pediatrics, University Hospital Münster, Münster, Germany; 35 Department of Pediatric Nephrology, Hôpital Robert Debré, Assistance Publique Hôpitaux de Paris, Paris, France; 36 Department of Nephrology, Edouard Herriot Hospital, Rare Disease Reference Center (MAREGE), ORKID Network, Lyon, France; 37 Nephrology, Dialysis and Transplantation Unit, Department of Medicine, University of Padova, Padova, Italy; 38 Department of Nephrology, Research Institute of Medical Innovations, Radboud University Medical Centre, Nijmegen, The Netherlands; 39 Division of Nephrology, Department of Internal Medicine, Amsterdam University Medical Center, Amsterdam, The Netherlands; 40 Department of Pediatric Nephrology, Vall d’Hebron Universitary Hospital, Barcelona, Spain; 41 Faculty of Medical Sciences, Biosciences Institute, Newcastle University, Newcastle upon Tyne, UK; 42 Renal Services, The Newcastle upon Tyne Hospitals NHS Foundation Trust, Freeman Road, Newcastle upon Tyne, UK; 43 NIHR Newcastle Biomedical Research Centre, Newcastle Upon Tyne, UK; 44 Department of Pediatric Nephrology, University Hospital Toulouse, Toulouse, France; 45 Department of Nephrology, Ghent University Hospital, Ghent, Belgium; 46 Paediatric Nephrology, Great Ormond Street Hospital for Children, NHS Foundation Trust, London, UK; 47 Department of Medical Translational Sciences, University of Campania “Luigi Vanvitelli,”, Naples, Italy; 48 Biogem, Institute of Molecular Biology and Genetics, Ariano Irpino, Italy

**Keywords:** genetics, hypokalemia, hypomagnesemia, patient-reported outcomes, tubulopathy

## Abstract

**Introduction:**

Gitelman syndrome (GS) is a rare inherited salt-losing tubulopathy with limited clinical data.

**Methods:**

Surveys were conducted with GS physicians in Europe and patients with GS in the Netherlands to compare findings with the general population.

**Results:**

Data from 587 patients (25% pediatric) across 13 countries showed 93% were genotyped, with 94% having variants in *SLC12A3*. Children with GS were shorter and lighter than the general population, with lower bodyweight persisting into adulthood. The sex distribution was uneven, with more males in childhood and more females in adulthood. Patients with GS had the expected electrolyte disorders as well as significantly lower blood phosphate levels. Positive correlations were found between blood magnesium and potassium, and potassium and aldosterone. Physicians reported muscle cramps, salt craving, and muscle weakness as most common GS symptoms. Patients with GS scored worse than the general population in fatigue, physical, and cognitive function; and ranked salt craving and polydipsia-polyuria as the most severe symptoms. Symptom burden was higher in adult females and patients with lower blood magnesium. Treatment mainly consisted of potassium (94%) and magnesium (50%) supplementation. Potassium-sparing medication (used in 33%) slightly increased blood potassium levels (3.2 vs. 3.1 mmol/l). Adult patients with GS had a high prevalence of chondrocalcinosis (15%) and elevated blood cell counts (26%). Compared with the general population, adult patients with GS had lower rates of chronic kidney disease (CKD) and hypertension, a similar rate of diabetes, but a higher rate of albuminuria or proteinuria (28%).

**Conclusions:**

These findings provide new insights into GS, highlight disease burden, and suggest areas for future research.

## Graphical abstract

**Figure:**
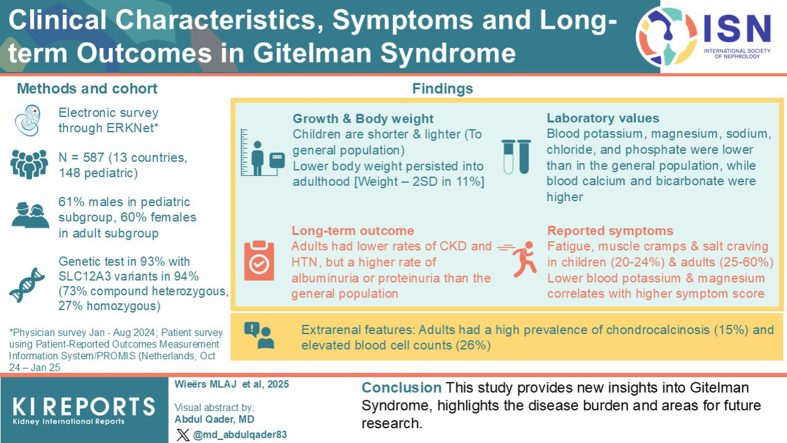

GS is an autosomal recessive salt-losing tubulopathy that is caused by genetic variants in *SLC12A3* leading to loss-of-function of the sodium-chloride cotransporter (NCC).^1^ Because GS impairs the overall function of the distal convoluted tubule, it leads to disturbances in sodium, chloride, potassium, magnesium, calcium and acid-base balance.^2^ Clinically, this is characterized by high urinary excretions of sodium, chloride, potassium, and magnesium leading to activation of the renin-angiotensin system, hypokalemia, metabolic alkalosis, and hypomagnesemia. Furthermore, GS is characterized by low urine calcium excretion, possibly due to increased calcium reabsorption in the proximal tubule.^3^^,^^4^ The symptoms of GS are related to fluid and electrolyte disturbances and include salt craving, polydipsia-polyuria, and muscle cramps.^5^ Calcium retention and hypomagnesemia can cause calcifications leading to chondrocalcinosis or choroidal calcifications.^6^^,^^7^

GS is a rare disease with an estimated prevalence of 1 in 40,000 people,^8^ although this may vary by ethnicity, with higher prevalence reported in Asian populations.^9^ Nevertheless, there is limited information on clinical characteristics, symptoms, and long-term outcomes. A Kidney Disease: Improving Global Outcomes controversy conference on GS identified several knowledge gaps, including effects on normal development, genotype-phenotype correlations, sex differences, patient-related outcomes; and long-term clinical outcomes, including the prevalence of CKD, proteinuria, hypertension and diabetes.^10^ Recently, 2 European surveys were conducted on 2 other tubulopathies—distal renal tubular acidosis and nephrogenic diabetes insipidus—revealing several new insights into physical and intellectual development, patient symptoms, and long-term outcomes.^11^^,^^12^ These studies illustrate the ability to obtain clinically relevant insights on rare diseases when combining data from multiple centers and countries.

The aim of this study was to study the clinical characteristics, symptoms, and long-term outcomes of GS using a European survey. Similar to the 2 previous surveys, we compared the data of patients with GS to the National Health and Nutrition Examination Survey (NHANES) data, which is a publicly available general population cohort.^13^ Because this cohort is from the United States, we also added a European reference population using the Rotterdam Study from The Netherlands.^14^ Furthermore, we conducted a patient survey focusing on patient-reported outcomes. Our study identified several novel aspects of GS with clinically relevant implications.

## Methods

### Physician and Patient Survey

Members of the European Reference Network for Rare Kidney Diseases were contacted via email to participate in an electronic survey to provide data on patients with a clinical or genetic diagnosis of GS. This survey was open from January 8 to August 1, 2024. A total of 66 questions were asked about demographics, genetics, laboratory values, extrarenal features, symptoms, and long-term outcomes (Physician Survey in the Supplementary Material). Regarding symptoms, we asked physicians to report whether patients experienced ≥ 1 GS-specific symptoms,^5^ as well as fatigue. In case of missing information or if provided data points were noted to be outliers, corresponding clinicians were contacted via e-mail for completion and/or verification of data. In addition to the physician survey, a patient survey was circulated among patients with GS in The Netherlands from October 1, 2024 to January 1, 2025 (Patient Survey in the Supplementary Materials). For this survey, we used the patient-reported outcomes measurement information system with questions on 6 domains.^15^ Furthermore, we asked patients to score GS-specific symptoms on a 5-point Likert scale.^5^ The physician and patient surveys were approved by the medical ethics committee of the Erasmus Medical Center (MEC-2023-0511). Physicians confirmed that patients signed informed consent through the European Reference Network for Rare Kidney Diseases, or that consent was obtained according to local regulations. Patients with GS participating in the patient survey signed informed consent. Electronic data capture was done with Castor EDC (The Netherlands).

### Definitions

We used the age of 18 years to separate the cohort into a pediatric and adult group. For children, height and weight were expressed as SD scores. These values were compared with reference data from the World Health Organization for height and the Centers for Disease Control for weight. For laboratory values, we asked physicians to specify the range in which the patient’s values were usually in to avoid bias because of intermittent outliers. Plasma renin and aldosterone were transformed to concentrations using established conversion factors.^16^^,^^17^ Based on physician-reported symptoms, we calculated a total symptom score. Kidney function was assessed using estimated glomerular filtration rate (eGFR) with the 2009 CKD-EPI equation, as recommended for European populations.^18^ For children, we used the Schwartz formula.^19^ The prevalence of CKD and degree of albuminuria were assessed using the Kidney Disease: Improving Global Outcomes classification.^20^ Hypertension was defined as blood pressure > 140/90 mm Hg for adults or > 95th percentile for children.^21^ Diabetes mellitus was defined as random glucose ≥ 11.1 mmol/l (200 mg/dl), fasting glucose ≥ 7.0 mmol/l (125 mg/dl) or the use of medication for diabetes mellitus.^22^

### Reference Populations

As control groups for the adult patients with GS, we used 2 cohorts from the general population, including NHANES from the United States (participants aged ≥ 18 years)^13^ and the Rotterdam Study from the Netherlands (participants aged ≥ 40 years).^14^ Both reference populations were used to compare height, weight, and body mass index with matching for age and sex. In addition, both reference populations were used for comparison of the laboratory data on electrolytes and acid-base balance except when data were available in only 1 cohort. The data on long-term outcomes were compared with the NHANES data because it allowed for better age-matching, except for albumin-to-creatinine ratio, which was only available in the Rotterdam Study.

### Statistics

Data were complete for physician- and patient-reported symptoms and ≥ 90% available for all other data, except for data on genetic variants (84%), height and body mass index in adults (85% and 84%, respectively), blood bicarbonate (84%), albuminuria or proteinuria (76%), plasma aldosterone (57%), and plasma renin (53%). Data were not imputed for analysis. Kolmogorov–Smirnov tests were performed to assess normality of the data. Data following a normal distribution were expressed as mean ± SD. Nonnormally distributed data were expressed as median with interquartile range. Differences between or within groups were analyzed using c2 or Fisher exact test, *t* test, Mann–Whitney U-test or 1-way analysis of variance with Tukey’s *post hoc* test, whichever was appropriate. Associations between dependent and independent variables were analyzed using linear regression. Matching between patients with GS and the reference populations was performed using the MatchIt package in R (version 4.3.1, R Foundation for Statistical Computing, Vienna, Austria) with a 1:3 case-control ratio via the optimal matching method. The patient survey data were analyzed using the patient-reported outcomes measurement information system which enables comparison with the general population using Cliff’s delta.^15^^,^^23^ All statistical analyses were performed using R.

## Results

### Demographics and Genetics

**Figure 1:**
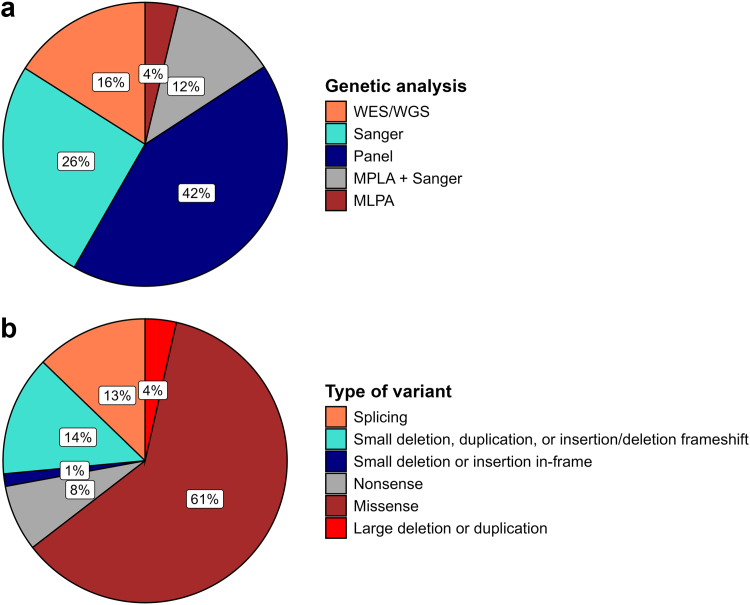
Distribution of genetic testing and identified genetic variants. (a) Out of the 545 patients who were genotyped, the type of genetic testing was specified in 506 patients (data availability 93%). (b) Out of the 514 patients in whom *SLC12A3* variant(s) were identified, the type of variant was specified in 424 patients (data availability 83%). MLPA, multiplex ligation-dependent probe amplification; WES, whole exome sequencing; WGS, whole genome sequencing.

A total of 587 patients with GS from 13 European countries were available for analysis, including 148 pediatric patients and 439 adult patients (Table 1, Supplementary Table S1). Sex was unevenly distributed with males being more prevalent in childhood (61%) and females in adulthood (60%, *P* < 0.05 for both). The average age at last follow-up was 12 years for pediatric patients and 39 for adult patients; the ages at diagnosis were 7 and 25 years of age, respectively. Females were older at diagnosis (23 ± 15 vs. 18 ± 15 years, *P* < 0.01). Reported ethnicities were predominantly White (> 80%), followed by Arab and Asian ethnicities (each ∼5%). Educational attainment in adult patients with GS was comparable to the average level across the European Union (Supplementary Table S2). Genetic testing was performed in > 90% of patients most often using a gene panel (42%), Sanger sequencing (26%), or whole exome or genome sequencing (16%) (Figure 1a). In the majority of patients, known pathogenic variants in *SLC12A3* were identified, usually in a compound heterozygous fashion. Most of the pathogenic variants constituted missense variants (61%) followed by nonsense variants and insertions–deletions (Figure 1b).

**Table 1:** Demographics and genetic data

Characteristics	All patients (*N* = 587)	Pediatric patients (*n* = 148)	Adult patients (*n* = 439)
Female sex, *n* (%)	323 (55)	57 (39)	266 (60)
Age at last follow-up (yrs)	32.4 ± 17.3	12.1 ± 3.9	39.3 ± 14.4
Age at diagnosis (yrs)	20.5 ± 14.8	7.2 ± 4.0	25.2 ± 14.3
Ethnicity			
Arab, *n* (%)	33 (6)	13 (9)	20 (5)
Asian	27 (5)	8 (5)	19 (4)
Black	1 (0.2)	1 (1)	0 (0)
Mixed	13 (2)	10 (7)	3 (1)
White	494 (84)	108 (73)	386 (88)
Not reported	19 (3)	8 (5)	11 (3)
Genotyped, yes (%)	545 (93)	147 (99)	398 (91)
*SLC12A3* variant(s) identified	514 (94)	144 (98)	370 (93)
Compound heterozygous	374 (73)	88 (61)	286 (77)
Homozygous	140 (27)	56 (39)	84 (23)
Consanguinity, *n* (%)	63 (11)	39 (26)	24 (5)

### Growth and Bodyweight

**Figure 2:**
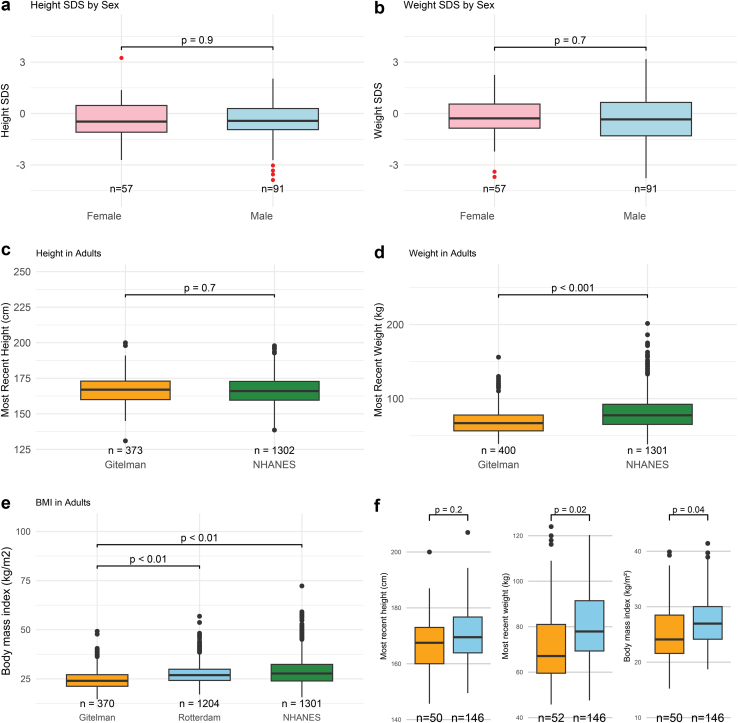
Height, weight, and body mass index in children and adults with Gitelman syndrome (GS). (a and b) In girls and boys with GS (a) height and (b) weight were analyzed using an SD score (SDS) based on data from the World Health Organization (height) or the Centers for Disease Control (weight). (c–e) In adult patients with GS, (c) height, (d) weight, and (e) body mass index (BMI) were compared with 1 or 2 age- and sex-matched reference populations, including the Rotterdam Study and National Health and Nutrition Examination Survey (NHANES). (f) To account for differences in height and weight between countries, height, weight, and BMI were compared between patients with GS from the Netherlands and healthy participants from the Rotterdam Study. Available data are reported below the graphs.

In children with GS, the median SD score for height and weight was < 0; however, in only 10 (7%) and 16 children (11%) this was < −2 SD score (Figure 2a and b). In adults with GS, the median height, weight, and body mass index were significantly lower than in 1 or both reference populations (Figure 2c and e). Next, we analyzed if the differences were caused by the Rotterdam Study reference group, because people from the Netherlands are relatively taller.^24^ When we restricted our comparison to Dutch patients with GS, height was no longer significantly different, but bodyweight and body mass index were still lower (Figure 2f).

### Laboratory Values

**Figure 3:**
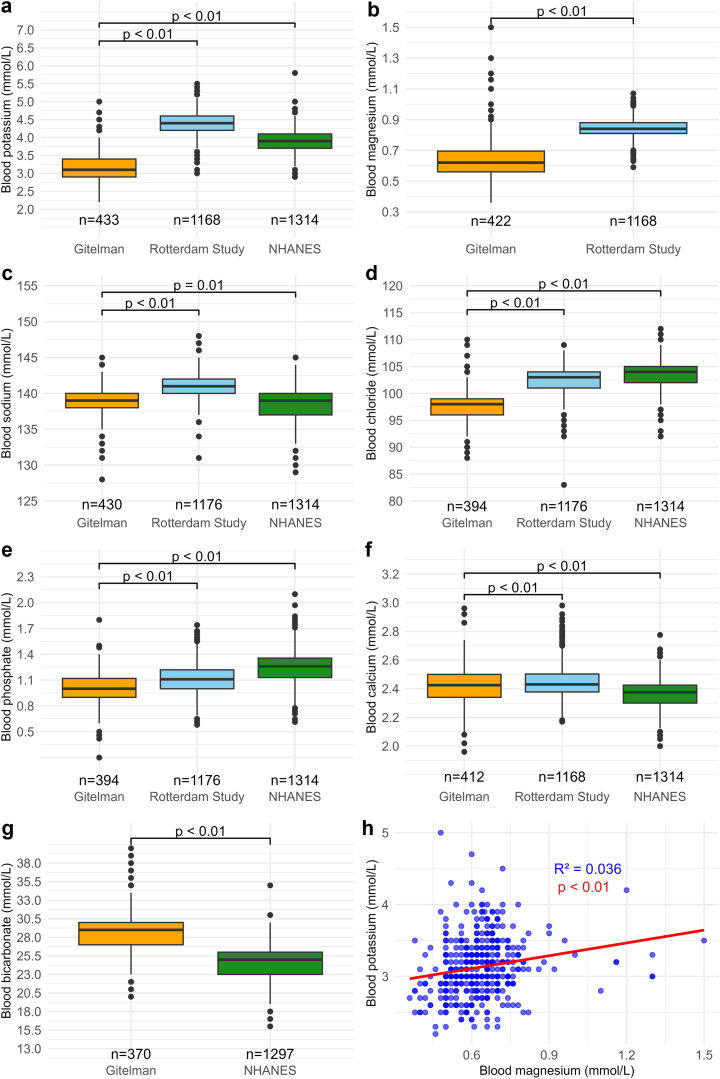
Laboratory values in adult patients with Gitelman syndrome. (a–g) Blood values for (a) potassium, (b) magnesium, (c) sodium, (d) chloride, (e) phosphate, (f) calcium, and (g) bicarbonate. Blood magnesium was only available in the Rotterdam Study, while blood bicarbonate was only available in the National Health and Nutrition Examination Survey (NHANES). Electrolytes and bicarbonate were measured in plasma (62%) or serum (38%) in the 549 patients for whom this was reported. Available data are reported below the graphs. Statistical analysis was performed using 1-way analysis of variance with Tukey’s *post hoc* test. (h) The association between blood magnesium and blood potassium was analyzed using linear regression; for this analysis data from 422 patients were available.

**Figure 4:**
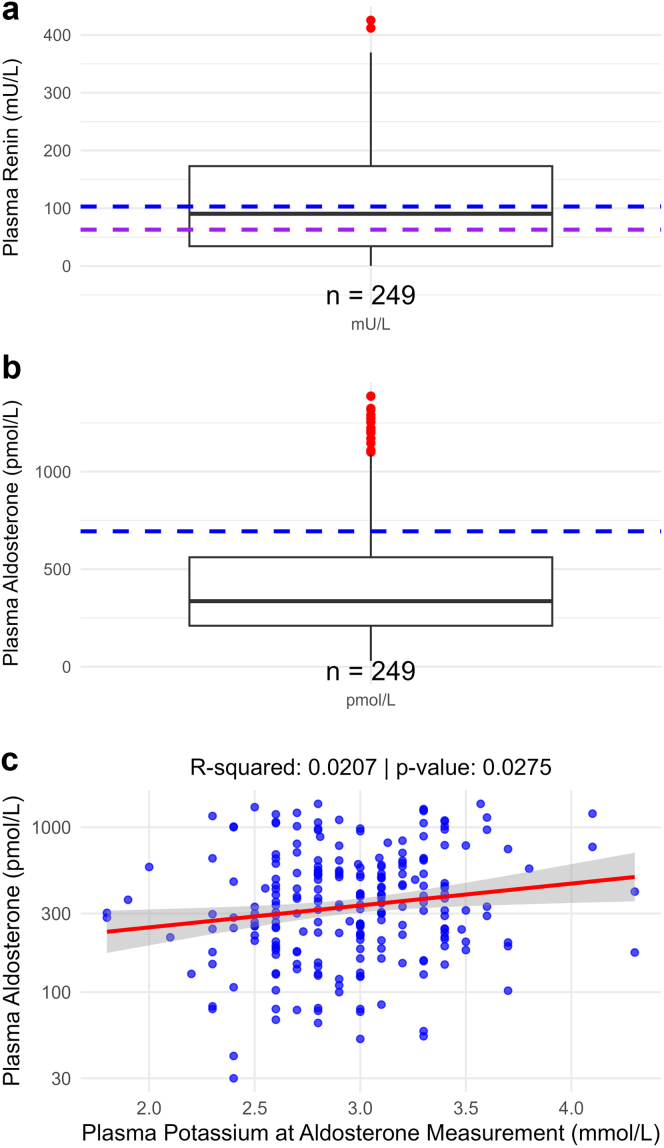
Plasma renin and aldosterone in children and adults with Gitelman syndrome. (a) The dashed lines indicate the upper levels of normal for plasma renin in males (blue, 103 mU/l) and females (purple, 63 mU/l). (b) The dashed line indicates the upper level of normal for plasma aldosterone (694 pmol/l). (c) The association between blood potassium and plasma aldosterone was analyzed using linear regression; for this analysis data from 247 patients were available. The available data are shown below the box plots. The renin and aldosterone data were converted to concentrations using established conversion factors.^16^^,^^17^

Adult patients with GS had significantly lower blood values for potassium, magnesium, sodium, chloride, and phosphate compared with 1 or both reference populations (Figure 3a–e). Conversely, blood values for calcium and bicarbonate were significantly higher than in 1 or both reference populations (Figure 3f and g). Blood magnesium and potassium levels were weakly correlated (Figure 3h). Blood magnesium levels were slightly lower in homozygous patients than in compound heterozygous patients (0.63 vs. 0.66 mmol/l, Supplementary Figure S1). Compared with male patients, female patients had higher blood chloride levels (98 ± 4 vs. 97 ± 3 mmol/l), but lower blood calcium (2.4 ± 0.1 vs. 2.5 ± 0.1 mmol/l) and bicarbonate levels (28 ± 3 vs. 29 ± 3 mmol/l, all *P* < 0.01). In children with GS, the values for electrolytes and bicarbonate were similar to adults, except for the expected higher phosphate levels (Supplementary Table S3). Plasma renin and aldosterone level were in the high range for plasma renin and in the normal range for plasma aldosterone (Figure 4a and b). Patients using nonsteroidal antiinflammatory drugs had lower plasma renin, but not aldosterone levels (Supplementary Table S4). We identified a significant but weak association between blood potassium and plasma aldosterone (Figure 4c). Urine calcium excretions were low (median: 1.1 mmol/d in 24 h urine and 0.06 mmol/mmol in spot urine, Supplementary Figure S2).

### Extrarenal Features

Chondrocalcinosis was reported in 0 of 147 pediatric patients and in 66 out of 435 adult patients (15%) (Table 2). Hyperparathyroidism was reported in 2 of 142 pediatric patients (1%) and in 11 of 434 adult patients (3%). One of these patients was hypercalcemic. Any elevated blood cell count was present in 18 of 146 pediatric patients (12%) and in 115 of 437 adult patients (26%). Erythrocytosis was reported with a similar frequency in pediatric and adult patients (7% for both), whereas thrombocytosis and leukocytosis were more common in adult patients (11 vs. 6% and 13 vs. 1%, respectively). The average hematocrit values for patients with erythrocytosis were 0.45 ± 0.03 l/l (children) and 0.50 ± 0.04 l/l (adults). The average thrombocyte and leukocyte counts were 460 ± 163 and 12.6 ± 2.1 × 10^9^/L (similar values for adults and children). The prevalence of elevated blood cell counts in patients using potassium-sparing medication was higher, although this did not reach statistical significance (Supplementary Table S5). Any bacterial infection and any allergy were reported in 10% and 12% of the patients, respectively; individual infections or allergies were reported in 1% to 10% of patients (Supplementary Table S6).

**Table 2:** Extra-renal features in Gitelman syndrome

Extrarenal feature	All patients^a^	Children^a^	Adults^a^
Chondrocalcinosis, *n*/total *N* (%)	66/582 (11)	0/147 (0)	66/435 (15)
Hyperparathyroidism, *n*/total *N* (%)	13/576 (2)	2/142 (1)	11/434 (3)
Any elevated blood cell count, *n*/total *N* (%)	133/583 (23)	18/146 (12)	115/437 (26)
• Erythrocytosis, *n* (%)	40 (7)	10 (7)	30 (7)
• Thrombocytosis, *n* (%)	55 (9)	9 (6)	46 (11)
• Leukocytosis, *n* (%)	59 (10)	1 (1)	58 (13)

a The total number of patients for whom these data were available was variable and therefore specified for each extrarenal feature (data availability 96%–100%).

### Physician- and Patient-Reported Symptoms

**Figure 5:**
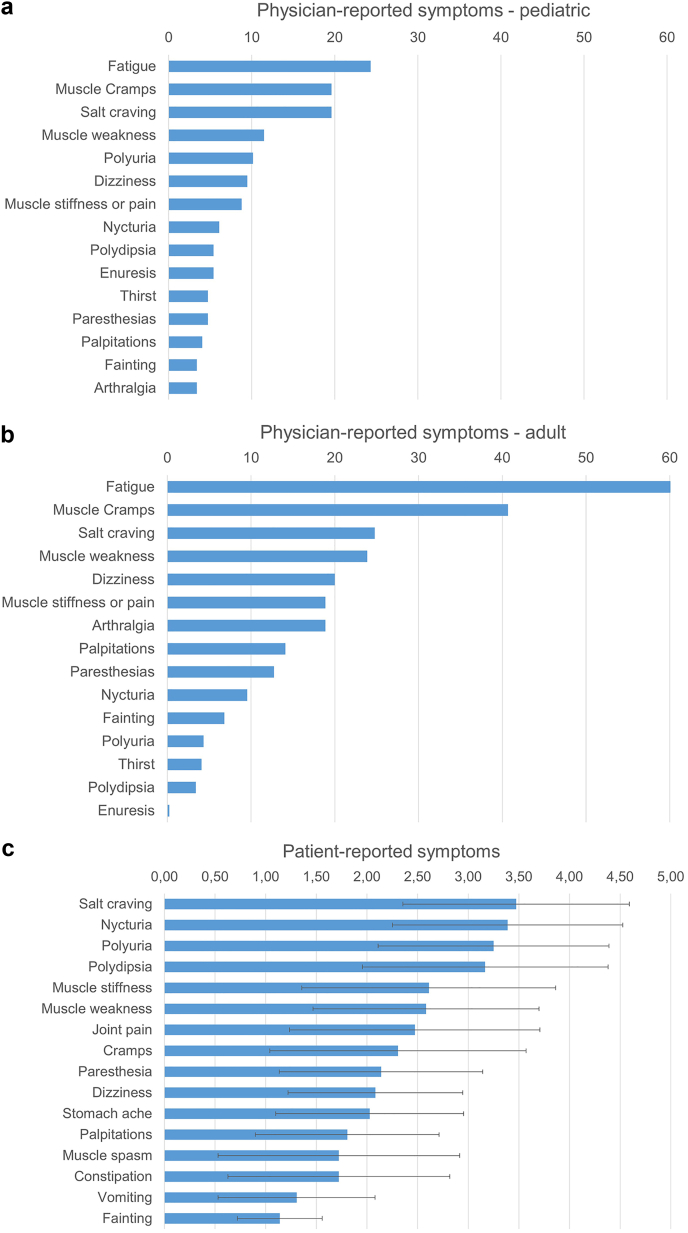
General and Gitelman syndrome-specific symptoms reported by physicians and patients. (a) The prevalence of symptoms is shown in percentages as reported by physicians for 148 children with Gitelman syndrome (GS). (b) The prevalence of symptoms is shown in percentages as reported by physicians for 439 adults with GS. (c) The burden of GS-specific symptoms (scale 0–5) as reported by 38 adult patients with GS from the Netherlands is shown as mean ± SD.

**Figure 6:**
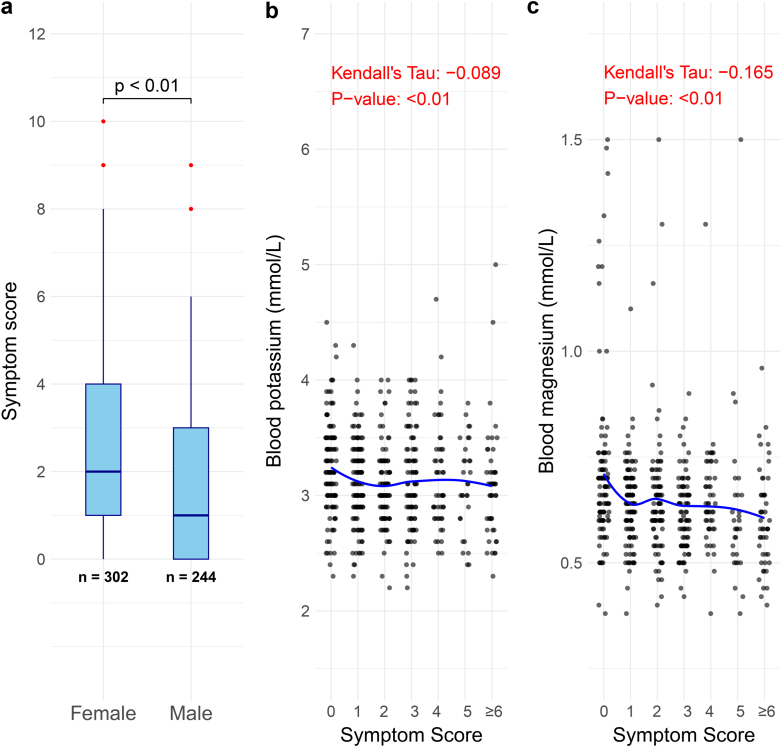
(a) Total symptom score in adult female and male patients with Gitelman syndrome. (b) Trend analysis between blood potassium levels and total symptom score. (c) Trend analysis between blood magnesium levels and total symptom score.

**Figure 7:**
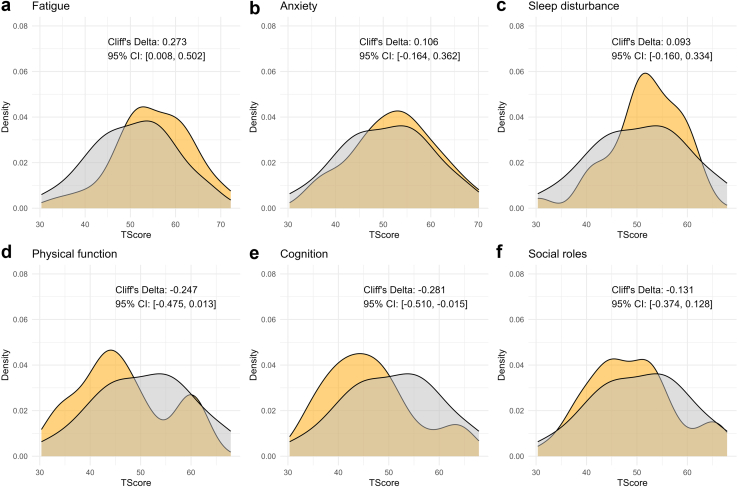
Results from the patient reported outcome measurement information system (PROMIS) on 6 domains in 38 adult patients with Gitelman syndrome (yellow) and the general population (grey). The domains include fatigue, anxiety, and sleep disturbance (for which higher T-score means that performance is worse than the general population) and physical function, cognitive function, and social roles (for which lower T-score means that performance is worse than the general population). Cliff’s delta is reported with 95% confidence intervals. Cliff’s delta was calculated using the effsize package in R (version 0.8.1, 2022).

Physicians reported fatigue, muscle cramps, and salt craving as the most common symptoms for children with GS with prevalences of 20% to 24% (Figure 5a). The same symptoms were most commonly reported in adults with GS, but with higher prevalences (fatigue: 60%, muscle cramps: 41%, salt craving: 25%, Figure 5b). The patient survey was distributed to 58 patients with GS in the Netherlands, with 38 patients (26 females, 12 males; age: 47 ± 15 years) completing it. In this analysis, salt craving, nycturia, polyuria, and polydipsia were identified as the most severe GS symptoms (average scores ≥ 3 on a 5-point scale, Figure 5c). The total symptom score was significantly higher in adult female patients with GS (Figure 6a). Furthermore, a significant trend was identified between lower blood potassium and magnesium levels and higher total symptom score (Figure 6b and c). The patient-reported outcomes measurement information system also enabled a standardized comparison of 6 domains between Patients with GS and the general population (Figure 7). This analysis showed that patients with GS performed significantly worse than the general population regarding fatigue, physical function, and cognitive function with a small to moderate effect-size (Cliff’s delta’s ∼ 0.25).

### Treatment

**Figure 8:**
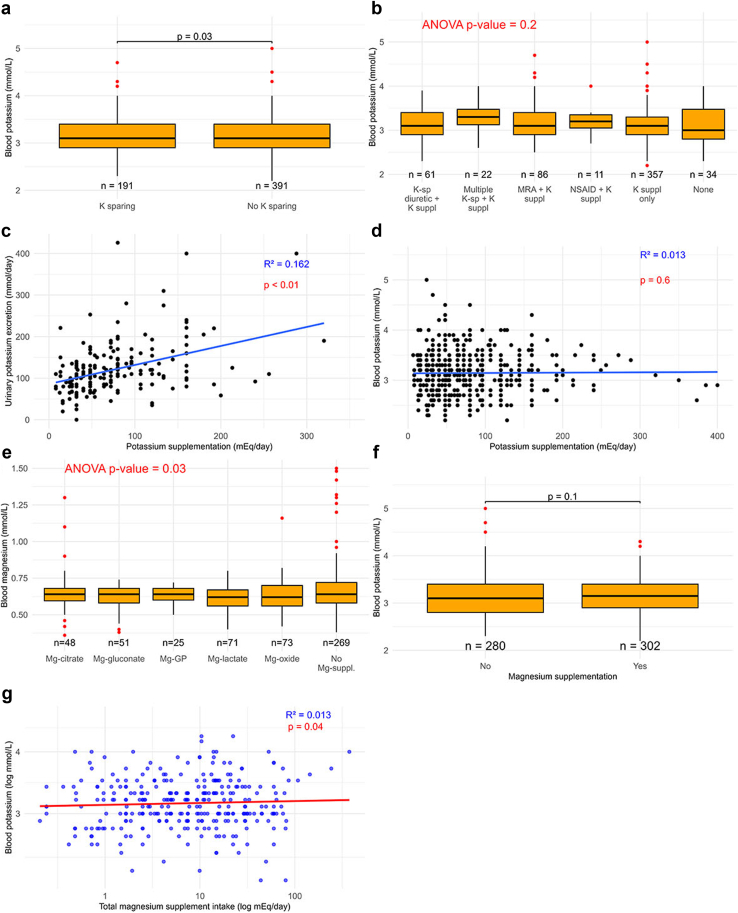
Laboratory values in relation to treatment in children and adults with Gitelman syndrome. (a) Blood potassium levels were compared in patients who did or did not use potassium-sparing diuretics. (b) Blood potassium levels were further analyzed by specific treatment regimens, including potassium-sparing (K-sp) diuretics, mineralocorticoid receptor antagonists (MRA), nonsteroidal antiinflammatory drugs (NSAIDs) and/or potassium supplementation (K suppl). (c) The association between the amount of potassium supplementation and urinary potassium excretion (*n* = 188). (d) The association between the amount of potassium supplementation and blood potassium (*n* = 537). (e) Blood magnesium levels were analyzed in patients who did or did not use magnesium (Mg) supplementation divided by the type of magnesium supplement, including Mg-citrate, Mg-gluconate, Mg-glycerophosphate (Mg-GP), Mg-lactate, and Mg-oxide. (f) Blood potassium levels were compared in patients who did or did not use magnesium supplementation. (g) The association between the amount of magnesium supplementation and blood magnesium was analyzed (*n* = 302). The data were analyzed using (a and f) *t* test, (b and e) 1-way analysis of variance with Tukey’s *post hoc* test, and (c, d, and g) linear regression.

Patients were treated with supplementation of potassium (94%), magnesium (50%), and sodium chloride (15%), potassium-sparing medications (33%), or combinations of these. Patients who used potassium-sparing medication had slightly but significantly higher blood potassium levels (3.2 vs. 3.1 mmol/l, Figure 8a). When the patients were divided further according to the main treatment combinations, we identified no significant differences in blood potassium levels (Figure 8b). We identified a positive association between the daily amount of potassium supplementation and 24-h urinary potassium excretion (Figure 8c), but not blood potassium level (Figure 8d). When blood magnesium levels were analyzed based on the type of magnesium supplementation or no supplementation, a significant difference was observed at the group level, but not between the subgroups (Figure 8e). Patients who used magnesium supplements did not have higher blood potassium (Figure 8f), but a higher daily magnesium supplementation was associated with a higher blood potassium level (Figure 8g).

### Long-Term Outcomes

**Figure 9:**
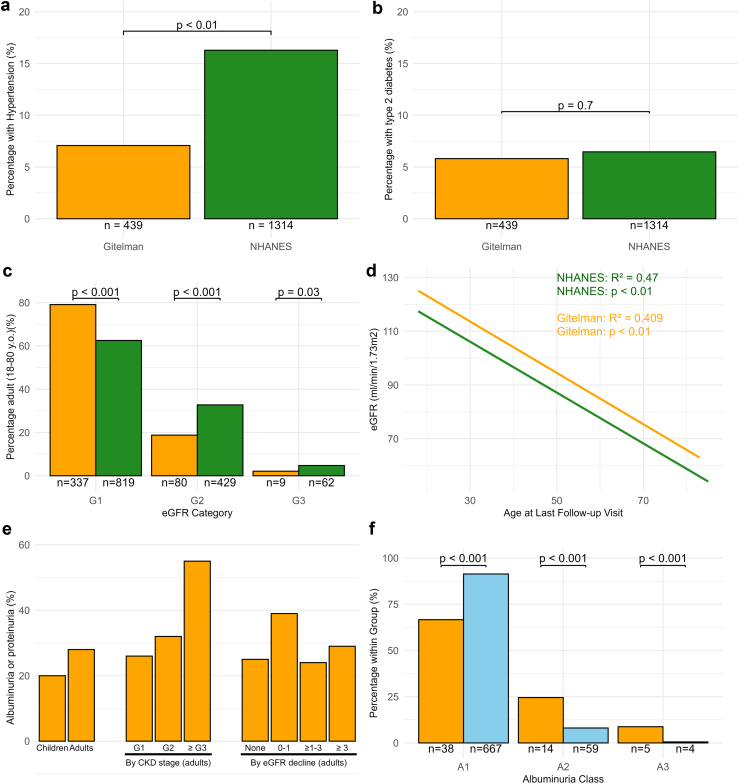
Long-term outcomes in adult patients with Gitelman syndrome. (a) Comparison of the prevalence of hypertension in Patients with GS who were age- and sex-matched to participants of the National Health and Nutrition Examination Survey (NHANES). (b) Comparison of the prevalence of type 2 diabetes mellitus in patients with GS who were age- and sex-matched to participants of the NHANES. (c) Distribution of chronic kidney disease (CKD) stages G1, G2, and G3 in patients with GS (orange) and age- and sex-matched participants of NHANES (green). (d) Association between age and estimated glomerular filtration rate (eGFR) at last follow-up in patients with GS (*n* = 430) and age- and sex-matched participants from the NHANES cohort (*n* = 1314). (e) Presence of albuminuria or proteinuria in children and adults with GS with further distribution in adults by CKD stage and eGFR decline (see also Table S5). (f) Comparison of urine albumin to creatinine ratios in adult Patients with GS (orange) and age- and sex-matched participants from the Rotterdam Study (blue). A1: < 3 mg/mmol, A2: 3–30 mg/mmol, A3: > 30 mg/mmol. The data were analyzed using (a, b, c, and f) *t* test or (d) linear regression.

Adult patients with GS were found to have a significantly lower prevalence of hypertension and a similar prevalence of diabetes mellitus when compared with the age- and sex-matched NHANES group (Figure 9a and b). Adult Patients with GS more often had normal kidney function and less often, stage G2 and G3 CKD (Figure 9c). When analyzing eGFR at the age of last follow-up, the relationship between eGFR and age ran at a higher level in patients with GS but followed a course that was parallel to the NHANES population (Figure 9d). Among the children with GS in this cohort, there was 1 child with hypertension, 1 child with CKD stage G3a, and no child with diabetes mellitus. Data on the assessment of albuminuria or proteinuria were available in 93% of children and 76% of adults with GS (Supplementary Table S7). Twenty percent of children and 28% of adults with GS had albuminuria or proteinuria in at least 1 measurement (Figure 9e). Adults with a higher CKD stage more often had albuminuria or proteinuria; however, the prevalence of albuminuria or proteinuria was not higher in those with a more rapid eGFR decline (Figure 9e, Supplementary Table S7). When compared with the Rotterdam Study, stage A2 and A3 albuminuria were significantly more common in adult patients with GS than in the general population (Figure 9f).

### Subgroup Analysis

Because GS was not genetically confirmed in all patients, we performed a subgroup analysis restricted to the genotyped patients (398/439 adults, 91%; 147/148 children, 99%). This analysis showed that the conclusions regarding height and bodyweight, laboratory values, extra-renal features, symptoms, treatment, and long-term outcomes remained unchanged, except for one association that became non-significant (the association between blood potassium and plasma aldosterone, r^2^ 0.015, *P*-value 0.06, Supplementary Table S8).

## Discussion

GS is a rare disease, and that complicates a full understanding of its disease burden, natural progression, and potential management strategies. The aim of this study was to analyze the clinical characteristics, symptoms, and long-term outcomes of GS through a European survey conducted among physicians treating these patients. This allowed us to establish the largest cohort of children and adults with GS reported to date. In addition, we conducted a patient survey to explore patient-reported outcomes related to both general and GS-specific symptoms. The findings offer several new insights into GS, which will be discussed below.

Growth in children with GS was below average; however, the mean height for most children fell within 2 SDs. This finding aligns with a previously reported small cohort of children with GS.^25^ In addition, no significant differences in height were observed in adults with GS compared with reference populations, suggesting catch-up growth. However, bodyweight was notably lower in adults with GS. Interestingly, in rats, potassium deficiency reduced food intake and inhibited muscle and bodyweight gain, potentially through its effect on insulin-like growth factor 1.^26^ This suggests that the lower bodyweight in patients with GS may be due to electrolyte imbalances or the disease’s impact on appetite. Previous studies have demonstrated that other tubulopathies, such as distal renal tubular acidosis and nephrogenic diabetes insipidus, can affect normal development in height and weight.^11^^,^^12^

The genetic analysis showed a distribution between compound heterozygous and homozygous variants of approximately 60% to 40% in children and approximately 75% to 25% in adults, with overall approximately 60% missense variants, which is in line with previous reports.^27^, ^28^, ^29^ We identified no clear genotype-phenotype correlations, except for slightly lower blood magnesium levels in homozygous patients. Previous studies in Asia did identify genotype-phenotype correlations, including lower blood chloride and potassium levels in patients with truncating variants,^28^ earlier age of onset and lower urine calcium excretion in homozygous patients,^30^ and lower blood magnesium levels in Japanese patients with a mutational hotspot.^29^ Analysis of these factors was not possible in this study because of the lack of variant-specific data.

The laboratory data in our study confirm the biochemical profile of GS and reveal some less well-known aspects. For example, blood phosphate levels were significantly lower in adult patients with GS than in the general population. This tendency toward hypophosphatemia has been observed in 2 previous smaller cohorts and was ascribed to renal phosphate wasting.^31^^,^^32^ It would be relevant to study if low blood phosphate levels contribute to symptoms in patients with GS. Another finding was that blood potassium was positively associated with plasma aldosterone. In the context of GS, this well-established physiological relationship implies that hypokalemia suppresses plasma aldosterone. This may explain the relatively normal plasma aldosterone levels in our cohort. Hypokalemia-induced suppression of aldosterone may contribute to hypomagnesemia, because aldosterone has been identified as a factor contributing to magnesium reabsorption in patients with GS.^33^ If so, this effect would be in addition to the established downregulation of the magnesium transporter TRPM6 observed in GS.^3^

Our study examined the extrarenal manifestations of GS. Chondrocalcinosis was reported in 15% of adult patients with GS, which is 2 to 3 times higher than the prevalence in the general population.^34^ The true prevalence of chondrocalcinosis is probably even higher because a previous prospective study with systematic screening for chondrocalcinosis detected it in 79% of the patients with GS.^7^ Another striking finding was the high prevalence of elevated blood counts. Isolated erythrocytosis has a prevalence of 0.3% in the general population,^35^ whereas it was reported in 7% of the patients with GS. High angiotensin II levels may be responsible for increasing erythropoietin levels in patients with GS.^36^^,^^37^ It is unclear if activation of the renin-angiotensin system explains the effect on platelets and leukocytes. A recently reported extrarenal manifestation of GS is a higher susceptibility to infectious and allergic disease, possibly mediated by impaired interleukin-17 responses.^38^ The rates of infectious and allergic diseases in our survey were not clearly higher than those in the general population, although underreporting may play a role because these aspects of the disease may not yet be systematically evaluated by physicians.

A unique aspect of our study is the availability of both physician- and patient-reported symptoms. This comparison revealed that physicians reported salt craving, polydipsia, polyuria, and nycturia in only approximately 20% and approximately 5% to 10% of patients, respectively; whereas patients scored this as the most severe symptoms. Fatigue was reported by physicians as the most prevalent symptom. In patients, this symptom was assessed more objectively by using the patient-reported outcomes measurement information system approach.^15^ Patients with GS had worse outcomes for fatigue as well as physical and cognitive function compared with the general population. These findings are largely in agreement with a previous survey among 50 patients with GS, which showed a high prevalence of salt craving, nycturia, and fatigue as well as significantly lower scores for physical and emotional health than healthy subjects using the RAND-36 health-related quality-of-life questionnaire.^5^ In our study, the total symptom score based on the physician-reported symptoms was higher for adult females than for males. Possible explanations are that women with GS have a higher disease burden or express these symptoms more clearly to their physicians. Women with GS are therefore more likely to be diagnosed than men, which may account for the higher prevalence of adult female patients with GS in our cohort. In childhood, boys, however, seem to be more severely affected. Male patients presented at a younger age, which was previously observed in a single-center cohort of 101 patients with GS, where 47% of males and 26% of females presented before the age of 18 years.^39^ In this previous study, females with GS had less severe electrolyte disorders, which is similar to what we observed. Male sex in combination with the nature of the *SLC12A3* variant may determine GS severity.^40^ Female rats have higher abundances of total and phosphorylated NCC^41^ and NCC is enhanced by estrogen.^42^ Possibly, these sex differences in NCC regulation allow female patients with GS to partially rescue the genetic NCC inhibition.

Our study captures the current management of GS in Europe, which involves supplementation of potassium, magnesium, and/or sodium, sometimes in combination with potassium-sparing medications. Our data do not allow firm conclusions on treatment in GS because of confounding by indication. However, a number of findings merit further consideration. For example, our data indicate that a higher dose of potassium supplementation was associated with a higher urinary potassium excretion but not with higher blood potassium levels. In contrast, both a higher blood magnesium level and a higher dose of magnesium supplementation were associated with a higher blood potassium level, although the strength of these associations was weak. These observations support previous studies showing improvement of blood potassium with magnesium supplementation.^43^^,^^44^ This is relevant because we found that lower blood magnesium levels were more strongly associated with a higher symptom burden than lower blood potassium levels. Patients receiving potassium-sparing drugs had a slightly but significantly higher blood potassium levels, which is consistent with the results from a previous randomized clinical trial.^45^ Future studies should investigate whether magnesium supplementation deserves a more prominent role in the treatment of GS, although it is important to prevent its gastrointestinal side effects. In addition, research should address whether potassium-sparing drugs improve patient-relevant outcomes beyond simply increasing blood potassium levels.

One of the specific aims of this cohort was to investigate the long-term outcomes of GS. When compared with a matched reference population, we observed a significantly lower prevalence of hypertension and CKD, a similar prevalence of diabetes, and a significantly higher prevalence of albuminuria or proteinuria. A previous smaller study suggested that patients with GS develop hypertension with age despite its salt-losing phenotype^46^; however, we were unable to confirm this finding. Furthermore, abnormal glucose metabolism and insulin secretion have been reported in GS^47^; however, this did not translate into a higher prevalence of overt diabetes in our cohort. A recent study showed that the prevalence of kidney failure in patients with GS is very low (0.9%) and contrasts with other inherited tubulopathies (prevalence: 15%–90%).^48^ This is in line with our findings which demonstrate a lower prevalence of CKD and normal decline in eGFR with age. One caveat is that patients with GS often have lower bodyweight, which may result in an underestimation of CKD prevalence when using creatinine-based eGFR. Other inherited tubulopathies are frequently accompanied by hypercalciuria, which may explain why GS does not increase the risk of progressive loss of kidney function.^48^ Albuminuria and proteinuria, however, were very common in patients with GS with a prevalence similar to that in patients with diabetes with preserved kidney function.^49^ A previous smaller study identified a similar prevalence of proteinuria and linked this to higher angiotensin II levels.^50^ Nine of the patients in this previous study were biopsied showing hypertrophy of the juxtaglomerular apparatus, tubulointerstitial and intrarenal vascular changes, and glomerulopathy. Another report of a kidney biopsy with electron microscopy showed variability in glomerular basement membrane thickness and podocyte foot effacement.^51^ GS therefore represents a unique form of kidney injury characterized by glomerular and tubulointerstitial damage that does not lead to progressive CKD. The contribution of hypokalemia and the activated renin-angiotensin system to kidney injury in GS remains to be determined.^52^ Considering the possible role of angiotensin II, it would be interesting to study the effects of angiotensin receptor blockers on proteinuria in GS, potentially improving hypokalemia and erythrocytosis as well.^53^

The strengths of our study are the large sample size, the use of 2 reference populations, and the combination of a physician- and patient survey. The most important limitation of this study is that the physician survey was a retrospective cross-sectional cohort review with data captured via an online form. This emphasizes the need for a comprehensive international registry to collect longitudinal granular data on this rare disorder, as is currently being done in a European Reference Network for Rare Kidney Diseases subregistry. Another limitation is that we did not collect data on the diagnostic triggers for GS, which can range from clear symptoms to incidental findings such as hypokalemia.

In conclusion, this European study on GS has revealed several new aspects of the disease, including lower bodyweight, sex differences, reduced blood phosphate levels, and a higher prevalence of albuminuria compared with the general population. Our study underscores the importance of the interaction between magnesium and potassium balance. In addition, the patient survey highlights the significant burden of disease, encompassing both general and GS-specific symptoms. Many of these findings have clinical implications and clearly establish a research agenda for future studies.

## Appendix

### List of the Collaborators of the Gitelman Survey Study

Pilar Auñón, Department of Nephrology, Hospital Universitario 12 de Octubre, Madrid, Spain.

Detlef Bockenhauer, Department of Paediatric Nephrology, Universitair Ziekenhuis (UZ) Leuven and Cellular and Molecular Physiology, Katholieke Universiteit Leuven, Leuven, Belgium

Peter J. Conlon, Department of Nephrology and Transplantation, Beaumont Hospital, Dublin, Ireland and Royal College of Surgeons in Ireland

Cosima Erhardt, Universitätsklinikum Leipzig, Leipzig, Germany

Sacha Flammier, Department of Pediatric Nephrology, Rheumatology and Dermatology, University Children's Hospital, Lyon, France.

Cristina Blázquez Gómez, Sección Nefrología Pediátrica, Servicio de Pediatría, Hospital Universitario 12 de Octubre, Madrid, Spain

Sercin Guven, Division of Pediatric Nephrology, Department of Pediatrics, School of Medicine, Marmara University, Istanbul, Turkey.

Gerlineke Hawkins, Department of Nephrology and Medical Intensive Care, Charité-Universitätsmedizin Berlin, Berlin, Germany.

Jan Halbritter, Department of Nephrology and Medical Intensive Care, Charité-Universitätsmedizin Berlin, Berlin, Germany.

Maria Herthelius, Division of Paediatrics, Department of Clinical Science, Intervention, and Technology, Karolinska Institutet, Stockholm, Sweden.

Alba Herreros, Servicio de Nefrología, Fundació Puigvert, Barcelona, Spain

Dragan Klaric, Department of Internal Medicine, Zadar General Hospital, Zadar, Croatia

Nóra Ledó, Department of Internal Medicine and Oncology, Semmelweis University, Budapest, Hungary.

Pierluigi Marzuillo, Department of Woman, Child, and General and Specialized Surgery, Università degli Studi della Campania 'Luigi Vanvitelli,' Naples, Italy.

Susanne Schäfer, Heidelberg Center for Pediatric Research, Heidelberg, Germany

Roland Schmitt, Department of Nephrology and Hypertension, Hannover Medical School, Hannover, Germany and Department of Nephrology and Hypertension, University Hospital Schleswig-Holstein, Kiel, Germany.

Malgorzata Stanczyk, Department of Pediatric, Immunology and Nephrology, Polish Mother's Memorial Hospital Research Institute, Łódź, Poland

Francesa Taroni, Pediatric Nephrology, Dialysis and Transplantation Unit Fondazione IRCCS Ca' Granda, Ospedale Maggiore Policlinico, Milan, Italy.

Nikola Zagorec, Department of Nephrology and Dialysis, Dubrava University Hospital, Zagreb, Croatia.

## Disclosure

All the authors declared no competing interests.

## Supplementary Material

Supplementary File (PDF) **Physician survey. Patient survey. Figure S1.** Blood magnesium in patients with compound heterozygous or homozygous variants. **Figure S2.** Urine calcium excretion. **Table S1.** European countries contributing to the survey. **Table S2.** Level of education. **Table S3.** Laboratory values for electrolytes and bicarbonate in children with Gitelman syndrome. **Table S4.** Plasma renin and aldosterone in patients with or without the use of nonsteroidal antiinflammatory drugs. **Table S5.** Prevalence of elevated blood cell counts in patients with or without potassium-sparing medication. **Table S6.** Infections and allergies. **Table S7.** eGFR, eGFR decline, and albuminuria or proteinuria. **Table S8.** Subanalysis including genotyped patients.
