# Supplementary material for: Clinical Characteristics, Symptoms, and Long-Term Outcomes in Gitelman Syndrome
Source: Kidney Int Rep. 2025 Sep 4;10(11):3967–83. doi: 10.1016/j.ekir.2025.09.006 (PMC12640036; doi:10.1016/j.ekir.2025.09.006)
Supplement: Supplementary File (PDF) — Physician survey. Patient survey. Figure S1. Blood magnesium in patients with compound heterozygous or homozygous variants. Figure S2. Urine calcium excretion. Table S1. European countries contributing to the survey. Table S2. Level of education. Table S3. Laboratory values for electrolytes and bicarbonate in children with Gitelman syndrome. Table S4. Plasma renin and aldosterone in patients with or without the use of nonsteroidal antiinflammatory drugs. Table S5. Prevalence of elevated blood cell counts in patients with or without potassium-sparing medication. Table S6. Infections and allergies. Table S7. eGFR, eGFR decline, and albuminuria or proteinuria. Table S8. Subanalysis including genotyped patients. [file mmc1.pdf]

Supplement

Clinical characteristics, symptoms and long-term outcomes in Gitelman syndrome

Wieërs *et al.*

Table of contents

Table S1 : European countries contributing to the survey

Table S2 : Level of education

Table S3 : Laboratory values for electrolytes and bicarbonate in children with Gitelman syndrome

Table S4 : Plasma renin and aldosterone in patients with or without the use of non-steroidal anti-inflammatory drugs

Table S5 **:** Prevalence of elevated blood cell counts in patients with or without potassium-sparing medication

Table S6 : Infections and allergies

Table S7 : eGFR, eGFR decline, and albuminuria/proteinuria

Table S8 : Subanalysis including genotyped patients

Figure S1 : Blood magnesium in patients with compound heterozygous or homozygous variants

Figure S2 : Urine calcium excretion

Table S1: European countries contributing to the survey

**Country**	**All patients**<br><br>(n = 587)	**Pediatric patients**<br><br>(n = 148)	**Adult patients**<br><br>(n = 439)
France, n (%)	214 (36)	45 (30)	169 (39)
United Kingdom, n (%)	87 (15)	19 (13)	68 (16)
Italy, n (%)	77 (13)	23 (16)	54 (12)
The Netherlands, n (%)	65 (11)	12 (8)	53 (12)
Germany, n (%)	54 (9)	19 (13)	35 (8)
Belgium, n (%)	51 (9)	9 (6)	42 (10)
Spain, n (%)	27 (5)	17 (11)	10 (2)
Croatia, n (%)	5 (0.9)	0 (0)	5 (1)
Hungary, n (%)	2 (0.3)	0 (0)	2 (0.5)
Sweden, n (%)	2 (0.3)	2 (1)	0(0)
Ireland, n (%)	1 (0.2)	0 (0)	1 (0.2)
Turkey, n (%)	1 (0.2)	1 (0.7)	0 (0)
Poland, n (%)	1 (0.2)	1 (0.7)	0 (0)

Table S2: Highest level of education

	**All patients** (n = 587)	**Pediatric patients** (n = 148)	**Adult patients*** (n = 439)
Primary school, n (%)	71 (12)	58 (39)	13 (3)
Secondary school, n (%)	175 (30)	72 (49)	103 (24)
Bachelor's or equivalent, n (%)	75 (13)	5 (3)	70 (16)
Master's or equivalent, n (%)	67 (11)	0 (0)	67 (15)
Doctorate or equivalent, n (%)	19 (3)	0 (0)	19 (4)
Unknown or not reported, n (%)	180 (31)	13 (9)	167 (38)

*The educational attainment for tertiary education (Bachelor, Master or Doctorate) in adult patients with Gitelman syndrome was 36% ([70+67+19]/439). This is comparable to 32.6% in the European Union. Note that the countries contributing to this GS cohort included several high-income economies. Source: Educational attainment statistics, https://ec.europa.eu/eurostat/statistics explained/index.php?title=Educational_attainment_statistics.

Table S3: Laboratory values for electrolytes and bicarbonate in children with Gitelman syndrome

	**Children** (n = 148)		**Adults** (n = 439)	
**Blood values**	**Data availability** n (%)	**Values**	**Data availability** n (%)	**Values**
Potassium, mmol/l	148 (100)	3.2 ± 0.4	433 (99)	3.1 ± 0.4
Magnesium, mmol/l	148 (100)	0.70 ± 0.2	422 (96)	0.63 ± 0.1
Sodium, mmol/l	147 (99)	139 ± 2	430 (98)	139 ± 2
Chloride, mmol/l	144 (97)	98 ± 3	394 (90)	98 ± 4
Phosphate, mmol/l	142 (96)	1.27 ± 0.2	394 (90)	1.01 ± 0.2
Calcium, mmol/l	145 (98)	2.49 ± 0.1	412 (94)	2.43 ± 0.1
Bicarbonate, mmol/l	145 (98)	28 ± 2	370 (84)	29 ± 3

Table S4: Plasma renin and aldosterone in patients with or without the use of non-steroidal anti-inflammatory drugs

	**NSAID use** (n = 14)	**No NSAID use** (= 235)	**P-value**
Plasma renin	21 (13, 81)	97 (42, 181)	< 0.01
Plasma aldosterone	444 (284, 713)	328 (203, 537)	0.2

Medians with IQR are shown.

Table S5. Prevalence of elevated blood cell counts in patients with or without potassium-sparing medication

	**Patients using potassium-sparing medication** (n = 191)	**Patients not using potassium-sparing medication** (n = 391)	**P-value**
Erythrocytosis, n (%)	18 (9)	22 (6)	0.1
Thrombocytosis, n (%)	25 (13)	30 (8)	0.08
Leukocytosis, n (%)	26 (14)	33 (8)	0.06

Table S6: Infections and allergies

**Infections and allergies**	**Data available for 548 patients (93% of total patients)**
Any bacterial infection*, n (%)	53 (10)
Any history of abcess, n (%)	15 (3)
Recurrent upper respiratory tract infection, n (%)	31 (6)
Recurrent urinary tract infection, n (%)	33 (6)
Recurrent vaginal or oral candidiasis, n (%)	7 (1)
Eczema or dermatitis, n (%)	38 (7)
Any allergic disease*, n (%)	65 (12)
Contact or environmental allergy, n (%)	53 (10)
Food allergy, n (%)	23 (4)
Drug allergy, n (%)	40 (7)

*Patients were classified as having 'any bacterial infection' or 'any allergic disease' if they had at least one individual bacterial infection or allergic condition, respectively.

Table S7: eGFR, eGFR decline, and albuminuria/proteinuria

**Category**	**Subcategory**	**Total number**	**Presence of albuminuria or proteinuria** n (%)
Children	All patients	137	28 (20)
Adults	All patients	332	93 (28)
CKD stage	G1	254	66 (26)
	G2	65	21 (32)
	≥ G3	11	6 (55)
eGFR decline	No decline	187	47 (25)
	0 to 1 ml/min per 1.73m^2/year	76	30 (39)
	>1 to 3 ml/min per 1.73m^2/year	41	10 (24)
	> 3 ml/min per 1.73m^2/year	14	4 (29)

Legend: See also Figure 8E. Data availability was as follows: albuminuria/proteinuria in children 137/148 (93%), in adults 332/439 (76%), CKD stage in adults 330/439 (75%), eGFR decline in adults 318/439 (72%).

Table S8: Subanalysis including genotyped patients.

Table S8A: Within group comparisons.

**Variable**	**Subgroup 1**	**Subgroup 2**	**P-value**	**Figure number full cohort**
Height SDS	Female<br>-0.4 ± 1.1<br>(n = 56)	Male<br>-0.4 ± 1.1<br>(n = 91)	0.94	2A
Weight SDS	Female<br>-0.3 ± 1.2<br>(n = 56)	Male<br>-0.4 ± 1.5<br>(n = 91)	0.7	2B
Symptom score	Female<br>2.7 ± 2.2<br>(n = 302)	Male<br>1.8 ± 1.7<br>(n = 244)	< 0.01	6A
Blood potassium	K-sparing<br>3.2 ± 0.42<br>(n = 175)	No K-sparing<br>3.1 ± 0.39<br>(n = 371)	< 0.05	8A
Blood potassium	No Mg supplementation<br>3.1 ± 0.4<br>(n = 268)	Magnesium supplementation<br>3.2 ± 0.4<br>(n = 275)	0.08	8F

Table S8B: Comparisons with the general population

**Variable**	**GS**	**Rotterdam**	**NHANES**	**P-value GS vs. RS**	**P-value GS vs. NHANES**	**Figure number full cohort**
Height	166 ± 9.5 (n = 340)	N/A	167 ± 9.9 (n = 1183)	N/A	0.8	2C
Weight	68.5 ± 15.9 (n = 366)	81.5 ± 17.3 (n = 1093)	80.4 ± 21.4 (n = 1182)	P < 0.01	P < 0.01	2D
BMI	24.6 ± 4.95 (n = 337)	27.6 ± 4.95 (n = 1093)	28.9 ± 7.02 (n = 1182)	P < 0.01	P < 0.01	2E
Height – Netherlands	168 ± 9.66 (n = 50)	170 ± 9.46 (n = 146)	N/A	P = 0.2	N/A	2F
Weight – Netherlands	73 ± 19.4 (n = 52)	79.9 ± 15.7 (n = 146)	N/A	P < 0.01	N/A	2F
BMI - Netherlands	25.6 ± 5.64 (n = 50)	27.5 ± 4.72 (n = 146)	N/A	P < 0.01	N/A	2F
Blood potassium	3.1 ± 0.4 (n = 395)	4.4 ± 0.3 (n = 1063)	3.9 ± 0.3 (n = 1194)	P < 0.01	P < 0.01	3A
Blood magnesium	0.63 ± 0.13 (n = 386)	0.84 ± 0.06 (n = 1063)	N/A	P < 0.01	N/A	3B
Blood sodium	139 ± 2.14 (n = 393)	141 ± 1.81 (n = 1070)	139 ± 2.15 (n = 1194)	P < 0.01	P < 0.01	3C
Blood chloride	97.7 ± 3.06 (n = 361)	102 ± 2.17 (n = 1070)	104 ± 2.63 (n = 1194)	P < 0.01	P < 0.01	3D

Blood phosphate	1.01 ± 0.19 (n = 379)	1.11 ± 0.17 (n = 1063)	1.25 ± 0.18 (n = 1194)	P < 0.01	P < 0.01	3E
Blood calcium	2.43 ± 0.13 (n = 379)	2.45 ± 0.11 (n = 1063)	2.37 ± 0.09 (n = 1194)	P < 0.05	P < 0.01	3F
Blood bicarbonate	28.7 ± 2.7 (n = 342)	N/A	24.5 ± 2.2 (n = 361)	N/A	P < 0.01	3G
Plasma renin	116 ± 99.8 (n = 239)	N/A	N/A	N/A	N/A	4A
Plasma aldosterone	330 ± 315 (n = 237)	N/A	N/A	N/A	N/A	4B
Hypertension	7.8%	N/A	16.6%	N/A	P < 0.01	9A
Diabetes	6.0%	N/A	6.4%	N/A	0.97	9B
CKD - G1	81%	N/A	63%	N/A	P < 0.01	9C
CKD - G2	18%	N/A	31%	N/A	P < 0.01	9C
CKD - G3	1.3%	N/A	5%	N/A	P < 0.01	9C
CKD – A1	67%	92%	N/A	P < 0.01	N/A	9F
CKD – A2	24%	7.5%	N/A	P < 0.01	N/A	9F
CKD – A3	9%	0.5%	N/A	P < 0.01	N/A	9F

Table S8C: Associations

**Dependent variable**	**Independent variable**	**R^2**	**P-value**	**Figure number full cohort**
Blood potassium	Blood magnesium	0.04	< 0.01	3H
Plasma aldosterone	Blood potassium at aldosterone measurement	0.015	0.06	4C
Blood potassium	Symptom score	-0.164	< 0.01	6B
Blood magnesium	Symptom score	-0.089	0.005	6C
Urinary potassium	Potassium supplementation	0.128	< 0.01	8C
Blood potassium	Potassium supplementation	0.001	0.8	8D
Blood potassium	Magnesium supplementation	0.04	< 0.01	8G
eGFR	Age at last follow-up	GS: 0.432<br>NHANES: 0.48	GS: < 0.01<br>NHANES: < 0.01	9D

Table S8D: Multiple group comparisons

**Parameter**	**Subgroups**						**P-value (ANOVA)**	**Figure**
Blood potassium	K-sp + K suppl<br><br>3.2 ± 0.5 (n = 75)	Multiple K-sp + K suppl<br><br>3.3 ± 0.4 (n = 20)	MRA + K suppl<br><br>3.1 ± 0.4 (n = 59)	NSAID + K suppl<br><br>3.2 ± 0.4 (n = 11)	K suppl only<br><br>3.1 ± 0.4 (n = 338)	None<br><br>3.1 ± 0.5 (n = 30)	0.15	8B
Blood magnesium	Citrate<br><br>0.65 ± 0.12 (n = 33)	Gluconate<br><br>0.63 ± 0.08 (n = 48)	GP<br><br>0.63 ± 0.06 (n = 25)	Lactate<br><br>0.61 ± 0.08 (n = 69)	Oxide<br><br>0.63 ± 0.11 (n = 68)	None<br><br>0.67 ± 0.18 (n = 259)	0.03	8E

Figure S1: Blood magnesium in patients with compound heterozygous or homozygous variants

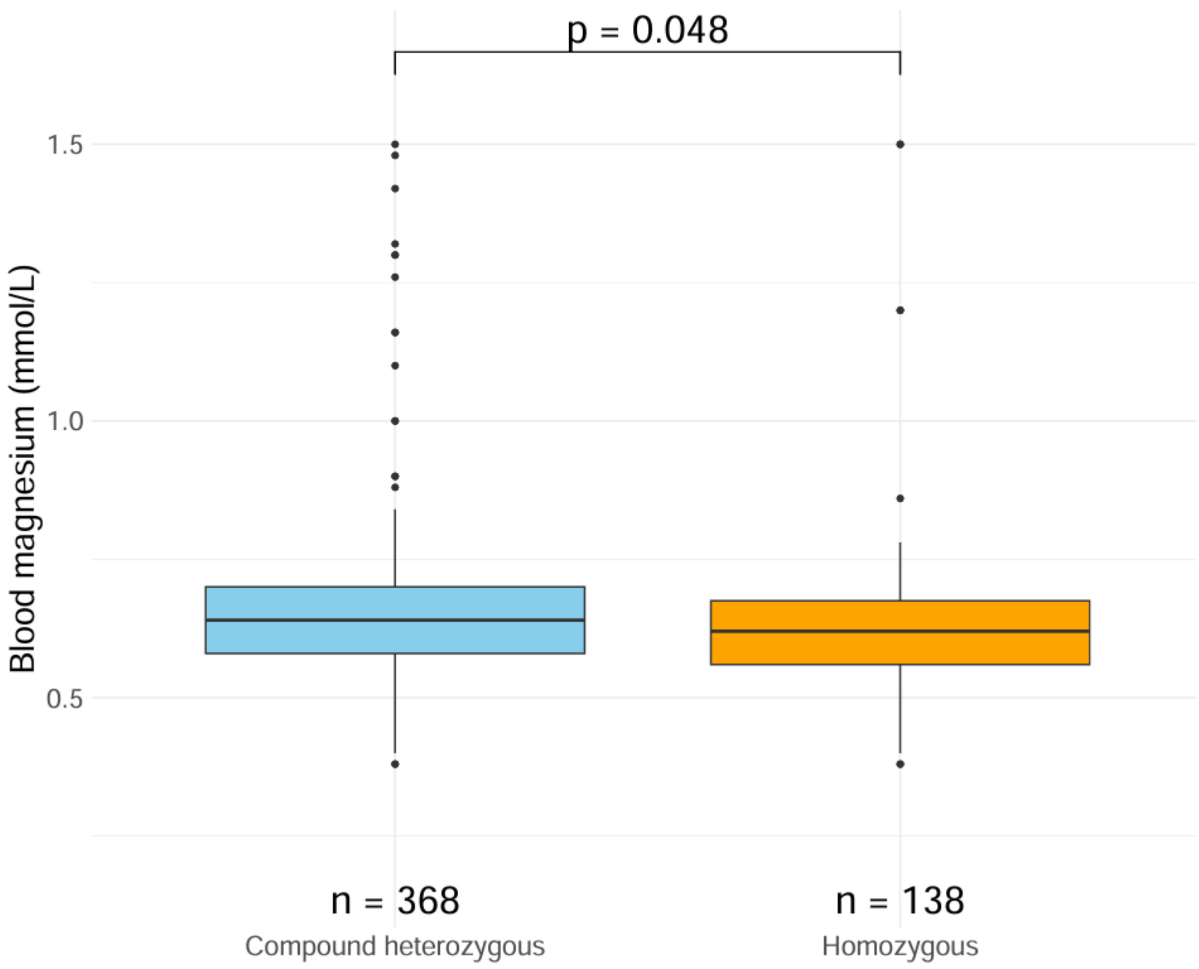

Figure S2: Urine calcium excretion.

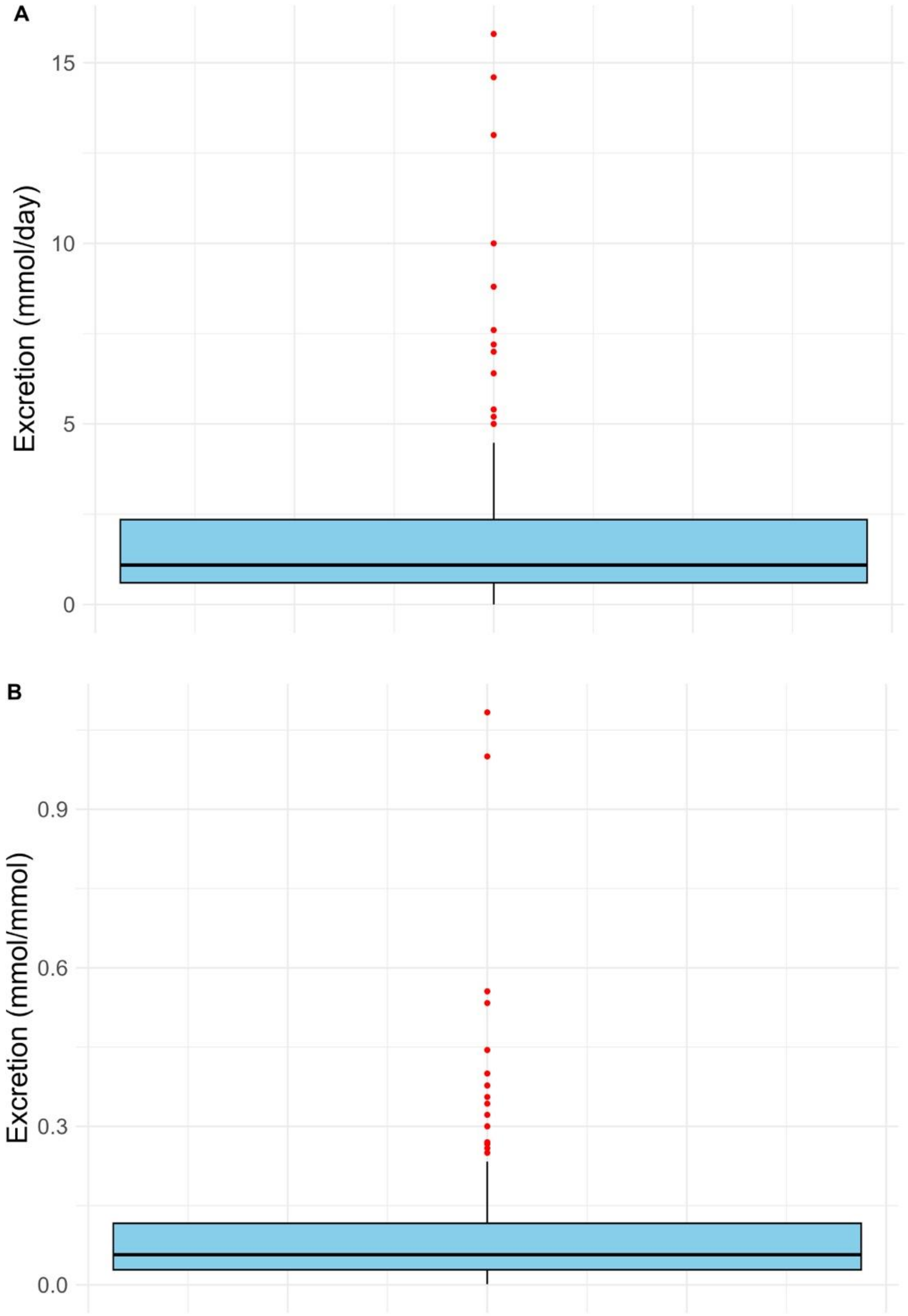

Legend:

(A) Urine calcium was measured in 24h urine in 158 patients

(B) Urine calcium was measured in spot urine in 186 patients.

Surveys of Gitelman Survey Study - version 110.61

Printed on 06-03-2025 10:32:31 by Michiel Wieers

Survey 'Gitelman Survey Study'

Gitelman Survey Study - Permission and identifyer of clinician

Number	Question	Answers
	Thank you for taking the time to fill in our survey! Please remember that this survey is meant for only 1 patient and is best filled in during one sitting. For each new patient please click the link again and complete the survey.	
1.1	Please confirm that you have permission or a waiver to enter anonymized data in this survey.	◯ Confirmed
1.2	Please fill in your first + last name and the institution you work for	
1.3	Please write your email adress, we might need this to contact you in case of significant outliers	

Gitelman Survey Study - Demographics

Number	Question	Answers
2.1	Sex	◯ Female<br>◯ Male
2.2	Age at last follow-up visit + year	**Age at last follow-up visit** ___ **Year of last follow-up visit** ___
2.3	Age at clinical or genetic diagnosis (whichever came first)	___ Years
2.4	Ethnicity	◯ White<br>◯ Arab<br>◯ Asian<br>◯ Black<br>◯ Mixed
2.5	Most recent height	___ cm
2.6	Most recent body weight	___ kg

2.7	Highest level of education	◯ Primary school<br>◯ Secondary school<br>◯ Bachelor's or equivalent<br>◯ Master's or equivalent<br>◯ Doctorate or equivalent<br>◯ Unknown
2.8	Please add any additional comments you may have	

Gitelman Survey Study - Genotype

Number	Question	Answers
3.1	Was the patient genotyped?	◯ Yes<br>◯ No
3.1.1	***If 'Was the patient genotyped?' is equal to 'Yes' answer this question:***<br>Which genetic analysis was used?	◯ Panel<br>◯ WES/WGS<br>◯ MLPA<br>◯ Sanger<br>◯ MPLA + Sanger
3.1.2	***If 'Was the patient genotyped?' is equal to 'Yes' answer this question:***<br>Please specify if homozygous or compound heterozygous	◯ Homozygous<br>◯ Compound heterozygous
3.1.3	***If 'Was the patient genotyped?' is equal to 'Yes' answer this question:***<br>Known pathogenic variant(s) in SLC12A3?	◯ Yes<br>◯ No
3.1.3.1	***If 'Known pathogenic variant(s) in SLC12A3?' is equal to 'Yes' answer this question:***<br>Please specify the type of pathogenic variant	◯ Missense<br>◯ Small deletion, duplication, or insertion/deletion frameshift<br>◯ Splicing<br>◯ Large deletion or duplication<br>◯ Nonsense<br>◯ Small deletion or insertion in-frame
3.2	Are the parents consanguineous?	◯ Yes<br>◯ No<br>◯ Unknown

3.3	Please add any additional comments you may have	

Gitelman Survey Study - Electrolytes and acid-base

Number	Question	Answers
	Please fill in the values for blood and urine electrolytes, if no data is available please check the box below the slider.	
4.1	Does your center measure electrolytes in serum or in plasma?	○ Serum<br>○ Plasma<br>○ Unknown
4.2	Average serum/plasma potassium (mmol/L)	1 (1) — 6 (6) mmol/L
4.3	.	☐ No data available serum/plasma potassium
4.4	Average serum/plasma magnesium (mmol/L)	0.2 (0.2) — 1.5 (1.5) mmol/L
4.5	.	☐ No data available serum/plasma magnesium
4.6	Average serum/plasma sodium (mmol/L)	110 (110) — 160 (160) mmol/L
4.7	.	☐ No data available serum/plasma sodium
4.8	Average serum/plasma chloride (mmol/L)	70 (70) — 120 (120) mmol/L
4.9	.	☐ No data available serum/plasma chloride
4.10	Average serum/plasma calcium (mmol/L), **(we assume albumin is normal)**	1.50 (1.5) — 3.50 (3.5) mmol/L
4.11	.	☐ No data available serum/plasma calcium
4.12	Average serum/plasma phosphate (mmol/L)	0.20 (0.2) — 2.20 (2.2) mmol/L

4.13	.	☐ No data available serum/plasma phosphate
4.14	Average serum/plasma bicarbonate (mmol/L)	20 (20) ——— 40 (40) mmol/L
4.15	.	☐ No data available serum/plasma bicarbonate
	If 24-h urine collection was performed, please specify urine electrolyte levels with all measurements from one collection (for spot urine, see below)	
4.16	Sodium (mmol/day)	(0) ——— 400 (400) mmol/day
4.17	Chloride (mmol/day)	(0) ——— 400 (400) mmol/day
4.18	Potassium (mmol/day)	(0) ——— 400 (400) mmol/day
4.19	Calcium (mmol/day)	(0) ——— 20 (20) mmol/day
4.20	Calcium (mg/day)	25 (25) ——— 300 (300) mg/day
	If spot urine was performed, please specify urine electrolyte levels with all measurements from one collection for spot urine (please enter concentrations, we will calculate the ratio with creatinine)	
4.21	Sodium (mmol/L)	10 (10) ——— 200 (200) mmol/L
4.22	Chloride (mmol/L)	20 (20) ——— 600 (600) mmol/L
4.23	Potassium (mmol/L)	10 (10) ——— 150 (150) mmol/L
4.24	Calcium (mmol/L)	0.01 (0.01) ——— 1 (1) mmol/L
4.25	Calcium (mg/dL)	0.01 (0.01) ——— 1 (1) mg/dL
4.26	Creatinine (mmol/L)	(0) ——— 25 (25) mmol/L

4.27	Creatinine (mg/dL)	(0) 2 (2) mg/dL
4.28	Please add any additional comments you may have	

Gitelman Survey Study - Plasma renin and aldosterone

Number	Question	Answers
5.1	Was plasma renin or aldosterone ever measured?	◯ Yes<br>◯ No
5.1.1	***If 'Was plasma renin or aldosterone ever measured?' is equal to 'Yes' answer this question:***<br>Use of diuretics or ACE-i/ARB during renin/aldo measurements?	◯ Yes<br>◯ No
5.1.2	***If 'Was plasma renin or aldosterone ever measured?' is equal to 'Yes' answer this question:***<br>Latest plasma renin (concentration or activity, please specify in unit)	**Value** **Unit**<br>Lastest plasma renin [ ] ---
5.1.3	***If 'Was plasma renin or aldosterone ever measured?' is equal to 'Yes' answer this question:***<br>Latest plasma aldosterone	**Value** **Unit**<br>Lastest plasma aldosterone [ ] ---
5.1.4	***If 'Was plasma renin or aldosterone ever measured?' is equal to 'Yes' answer this question:***<br>Blood potassium at the time of latest plasma aldosterone measurement	mmol/L
5.2	Please add any additional comments you may have	

Gitelman Survey Study - Drug treatment

Number	Question	Answers
6.1	Does your patient receive drug treatment (specifiaclly for Gitelman Syndrome)?	◯ Yes<br>◯ No

6.1.1	***If 'Does your patient receive drug treatment (specifiaclly for Gitelman Syndrome)?' is equal to 'Yes' answer this question:*** Amiloride (dose per day)	[ ] mg
6.1.2	***If 'Does your patient receive drug treatment (specifiaclly for Gitelman Syndrome)?' is equal to 'Yes' answer this question:*** Triamterene (dose per day)	[ ] mg
6.1.3	***If 'Does your patient receive drug treatment (specifiaclly for Gitelman Syndrome)?' is equal to 'Yes' answer this question:*** Spironolactone (dose per day)	[ ] mg
6.1.4	***If 'Does your patient receive drug treatment (specifiaclly for Gitelman Syndrome)?' is equal to 'Yes' answer this question:*** Eplerenone (dose per day)	[ ] mg
6.1.5	***If 'Does your patient receive drug treatment (specifiaclly for Gitelman Syndrome)?' is equal to 'Yes' answer this question:*** Indomethacin (dose per day)	[ ] mg
6.1.6	***If 'Does your patient receive drug treatment (specifiaclly for Gitelman Syndrome)?' is equal to 'Yes' answer this question:*** Proton pump inhibitor	○ Yes<br>○ No
6.1.6.1	***If 'Proton pump inhibitor' is equal to 'Yes' answer this question:*** Please specify the name and total daily dose	[ ]
6.1.7	***If 'Does your patient receive drug treatment (specifiaclly for Gitelman Syndrome)?' is equal to 'Yes' answer this question:*** Other drugs for treatment of GS (name and dose per day)	[ ]
6.2	Please add any additional comments you may have	[ ]

Gitelman Survey Study - Supplements

Number	Question	Answers

7.1	Does your patient take supplements?	◯ Yes<br>◯ No
7.1.1	***If 'Does your patient take supplements?' is equal to 'Yes' answer this question:***<br>Liquid potassium chloride	Liquid potassium chloride — **Dose (per day)** [ ] **Unit** [---]
7.1.2	***If 'Does your patient take supplements?' is equal to 'Yes' answer this question:***<br>Slow-release potassium chloride	Slow-release potassium chloride — **Dose (per day)** [ ] **Unit** [---]
7.1.3	***If 'Does your patient take supplements?' is equal to 'Yes' answer this question:***<br>Intravenous potassium chloride	Intravenous potassium chloride — **Dose** [ ] **Unit** [---] **Frequency (e.g. 1/week)** [ ]
7.1.4	***If 'Does your patient take supplements?' is equal to 'Yes' answer this question:***<br>Magnesium lactate	Magnesium lactate — **Dose (per day)** [ ] **Unit** [---]
7.1.5	***If 'Does your patient take supplements?' is equal to 'Yes' answer this question:***<br>Magnesium gluconate	Magnesium gluconate — **Dose (per day)** [ ] **Unit** [---]
7.1.6	***If 'Does your patient take supplements?' is equal to 'Yes' answer this question:***<br>Magnesium citrate	Magnesium citrate — **Dose (per day)** [ ] **Unit** [---]
7.1.7	***If 'Does your patient take supplements?' is equal to 'Yes' answer this question:***<br>Magnesium oxide	Magnesium oxide — **Dose (per day)** [ ] **Unit** [---]
7.1.8	***If 'Does your patient take supplements?' is equal to 'Yes' answer this question:***<br>Magnesium glycerophosphate	Magnesium glycerophosphate — **Dose (per day)** [ ] **Unit** [---]
7.1.9	***If 'Does your patient take supplements?' is equal to 'Yes' answer this question:***<br>Intravenous magnesium	Intravenous magnesium — **Dose** [ ] **Unit** [---] **Frequency (e.g. 1/week)** [ ]
7.1.10	***If 'Does your patient take supplements?' is equal to 'Yes' answer this question:***<br>Sodium chloride tablets	Sodium chloride tablets — **Dose (per day)** [ ] **Unit** [---]
7.1.11	***If 'Does your patient take supplements?' is equal to 'Yes' answer this question:***<br>Intravenous sodium chloride	Intravenous sodium chloride — **Dose** [ ] **Unit** [---] **Frequency (e.g. 1/week)** [ ]

7.1.12	***If 'Does your patient take supplements?' is equal to 'Yes' answer this question:*** Other supplements for treatment of GS, dose per day (please add unit)	
7.2	Please add any additional comments you may have	

Gitelman Survey Study - Hypertension

Number	Question	Answers
8.1	Does your patient have hypertension? (1-18 years >95th percentile, >18 years >140/90 mmHg with proper measurement technique)?	◯ Yes<br>◯ No
8.2	Was a 24-hour blood pressure measurement performed?	◯ Yes<br>◯ No
8.2.1	***If 'Was a 24-hour blood pressure measurement performed?' is equal to 'Yes' answer this question:*** Please fill in average day and night **systolic** blood pressure in mmHg	**Day** / **Night**<br>Average blood pressure
8.2.2	***If 'Was a 24-hour blood pressure measurement performed?' is equal to 'Yes' answer this question:*** Please fill in average day and night **diastolic** blood pressure in mmHg	**Day** / **Night**<br>Average blood pressure
8.3	Does your patient use anti-hypertensive drugs with hypertension as indication (i.e. not to raise plasma potassium)?	◯ Yes<br>◯ No
8.3.1	***If 'Does your patient use anti-hypertensive drugs with hypertension as indication (i.e. not to raise plasma potassium)?' is equal to 'Yes' answer this question:*** Please fill in which medication(s) + total daily dose of each	
8.4	Please add any additional comments you may have	

Gitelman Survey Study - Kidney function

Number	Question	Answers
9.1	Creatinine	**Value** **Unit**<br>Creatinine [ ] [--- ▾]
9.2	Age at time of last blood creatinine measurement	[ ] Years
9.3	If known, what is the average eGFR decline per year (in ml/min/1.73 m2)?	◯ No decline<br>◯ 0 to 1<br>◯ 1 to 2<br>◯ 2 to 3<br>◯ 3 to 4<br>◯ 4 to 5<br>◯ More than 5
9.4	Did your patient ever have proteinuria/albuminuria?	◯ Yes<br>◯ No<br>◯ Not measured
9.4.1	***If 'Did your patient ever have proteinuria/albuminuria?' is equal to 'Yes' answer this question:***<br>What was the highest albuminuria using **spot urine** (urine albumin to creatinine ratio, UACR)	◯ < 30 mg/g or < 3 mg/mmol<br>◯ 30-300 mg/g or 3-30 mg/mmol<br>◯ > 300 mg/g, > 30 mg/mmol<br>◯ Missing data
9.4.2	***If 'Did your patient ever have proteinuria/albuminuria?' is equal to 'Yes' answer this question:***<br>What was the highest proteinuria using **spot urine** (urine protein to creatinine ratio, UPCR)?	◯ < 150 mg/g (< 0.15 mg/mg) or < 15 mg/mmol<br>◯ 150-500 mg/g or (0.15-0.50 mg/mg) or 15-50 mg/mmol<br>◯ > 500 mg/g (> 0.50 mg/mg) or > 50 mg/mmol<br>◯ Missing data
9.4.3	***If 'Did your patient ever have proteinuria/albuminuria?' is equal to 'Yes' answer this question:***<br>What was the highest albuminuria using **24h urine?**	◯ < 30 mg/day<br>◯ 30-300 mg/day<br>◯ >300 mg/day<br>◯ Missing data
9.4.4	***If 'Did your patient ever have proteinuria/albuminuria?' is equal to 'Yes' answer this question:***<br>What was the highest proteinuria using **24h urine?**	◯ 0-300 mg/day<br>◯ >300 mg/day<br>◯ Missing data
9.5	Please add any additional comments you may have	[ ]

Gitelman Survey Study - Diabetes mellitus

Number	Question	Answers
10.1	Does your patient have type 1, type 2 or no diabetes?	◯ No diabetes<br>◯ Type 1 diabetes<br>◯ Type 2 diabetes<br>◯ Unknown
10.1.1	***If 'Does your patient have type 1, type 2 or no diabetes?' is not equal to 'No diabetes' answer this question:***<br>Please fill in which drugs for the treatment of diabetes are given + total daily dose of each	
10.2	Please add any additional comments you may have	

Gitelman Survey Study - Infection and immunity

Number	Question	Answers
11.1	Any bacterial infection recorded in medical history (e.g. chest X-ray proven pneumonia)?	◯ Yes<br>◯ No<br>◯ Unknown
11.2	Any previous history of an abcess (skin or glands, e.g. submandibular)?	◯ Yes<br>◯ No<br>◯ Unknown
11.3	Recurrent upper respiratory tract infection (≥3 in worst year) or requiring hospital admission?	◯ Yes<br>◯ No<br>◯ Unknown
11.4	Recurrent urinary tract infection (≥3 in worst year for females) or any in childhood or in males?	◯ Yes<br>◯ No<br>◯ Unknown

11.5	Recurrent vaginal (≥2 episodes) or oral candidiasis?	Yes<br>No<br>Unknown
11.6	Eczema/dermatitis?	Yes<br>No<br>Unknown
11.7	Any allergic disease?	Yes<br>No<br>Unknown
11.8	Contact/environmental allergy?	Yes<br>No<br>Unknown
11.9	Food allergy?	Yes<br>No<br>Unknown
11.10	Drug allergy?	Yes<br>No<br>Unknown
11.11	Please add any additional comments you may have	

Gitelman Survey Study - Joints, eyes, parathyroid and bone health

Number	Question	Answers
12.1	Does your patient have chondrocalcinosis?	Yes<br>No<br>Unknown

12.1.1	***If 'Does your patient have chondrocalcinosis?' is equal to 'Yes' answer this question:*** If yes, where predominantly?	☐ Ankles<br>☐ Spine<br>☐ Knee<br>☐ Wrist<br>☐ Jaw<br>☐ Shoulder<br>☐ Hip<br>☐ Elbow<br>☐ Eyes (schlerochoroidal)<br>☐ Site not mentioned
12.2	Is there a diagnosis of gout?	○ Yes<br>○ No<br>○ Unknown
12.3	What is the highest serum or plasma uric acid (if known)?	**Value** / **Unit (please fill in)**<br>Highest plasma uric acid level: ______ / ______
12.4	Is your patient using medication for chondrocalcinosis or gout?	○ Yes<br>○ No<br>○ Unknown
12.4.1	***If 'Is your patient using medication for chondrocalcinosis or gout?' is equal to 'Yes' answer this question:*** If yes, please specify which medication, type + dose.	______
12.5	Is there a diagnosis of hyperparathyroidism?	○ Yes<br>○ No<br>○ Unknown
12.6	If known, what is the bone mineral density of your patient?	○ Increased<br>○ Normal<br>○ Decreased
12.7	Does your patient have basal ganglia calcifications?	○ Yes<br>○ No<br>○ Unknown

12.8	Please add any additional comments you may have	

Gitelman Survey Study - Hematological

Number	Question	Answers
13.1	Did your patient ever have erythrocytosis/polycythemia?	◯ Yes<br>◯ No<br>◯ Not measured
13.1.1	***If 'Did your patient ever have erythrocytosis/polycythemia?' is equal to 'Yes' answer this question:***<br>Please fill in highest hematocrit value	Value / Unit<br>Hematocrit value [ ] [--- ▾]
13.1.2	***If 'Did your patient ever have erythrocytosis/polycythemia?' is equal to 'Yes' answer this question:***<br>If no hematocrit available, please fill in the highest hemoglobin level	Value / Unit<br>Hb value [ ] [--- ▾]
13.2	Did your patient ever have thrombocytosis?	◯ Yes<br>◯ No<br>◯ Not measured
13.2.1	***If 'Did your patient ever have thrombocytosis?' is equal to 'Yes' answer this question:***<br>Please specify highest value	[ ] x10^9/L
13.3	Did your patient ever have leukocytosis that was not explained by a clear intercurrent infection?	◯ Yes<br>◯ No<br>◯ Not measured
13.3.1	***If 'Did your patient ever have leukocytosis that was not explained by a clear intercurrent infection?' is equal to 'Yes' answer this question:***<br>Please specify highest value	[ ] x10^9/L
13.4	Was additional investigation done for haematological abnormalities?	◯ Yes<br>◯ No<br>◯ Not measured

13.4.1	***If 'Was additional investigation done for haematological abnormalities?' is equal to 'Yes' answer this question:*** Please specify (eg: JAK2 mutation testing or bone marrow biopsy)	
13.5	Please add any additional comments you may have	

Gitelman Survey Study - Performance score

Number	Question	Answers
14.1	Please estimate your patient's every day life performance score	◯ 100 - Normal to no complaints, no evidence of disease<br>◯ 90 - Able to carry on normal activity, minor signs or symptoms of disease<br>◯ 80 - Normal activity with effort, some signs or symptoms of disease<br>◯ 70 - Cares for self, unable to carry on normal activity or to do active work<br>◯ 60 - Requires occasional assistance, but is able to care for most personal needs<br>◯ 50 - Requires considerable assistance and frequent medical care<br>◯ 40 - Disabled, requires special care and assistance
14.2	Please specify the patient's symptoms (multiple may apply).	☐ Fatigue<br>☐ Dizziness<br>☐ Fainting<br>☐ Muscle weakness<br>☐ Cramps<br>☐ Muscle stiffness or pain<br>☐ Arthralgia<br>☐ Nycturia<br>☐ Polydipsia<br>☐ Polyuria<br>☐ Thirst<br>☐ Enuresis<br>☐ Salt craving<br>☐ Paresthesias<br>☐ Palpitations

14.3	Please specify any other notable items in the previous medical history	
14.4	Please add any additional comments you may have	

Gitelman Survey Study - Outro

Thank you for filling out our survey! If you wish to add more patients, please click the link in our email again to start again.

Surveys of Gitelman Survey Studie-patienten perspectief - version 53.51

Printed on 10-03-2025 11:22:56 by Michiel Wieers

Survey 'Gitelman Survey Study - Patienten Perspectief (kinderen)'

Gitelman Survey Study - Patienten Perspectief (kinderen) - Introduction

Beste deelnemer

Dankjewel om de tijd te nemen deze vragenlijst in te vullen! Het doel van onze studie is om een beter zicht te krijgen in hoe patienten met Gitelman syndroom hun ziekte ervaren. Dit doen we door een aantal stellingen over Gitelman syndroom (bijvoorbeeld de klachten) te bevragen in hoeverre jij je daarin kan vinden.

Het zou maximaal 30 minuten mogen duren om deze vragenlijst in te vullen. Je moet steeds een bolletje aanduiden wat jouw gevoel is ten aanzien van de stelling.

Alvast bedankt voor je bijdrage!

Groeten Het onderzoeksteam van de Gitelman Survey Studie

Gitelman Survey Study - Patienten Perspectief (kinderen) - Beschrijving

Number	Question	Answers
1.1	Wat is jouw geslacht?	◯ Man<br>◯ Vrouw
1.2	Wat is jouw leeftijd?	[ ] Jaar

Gitelman Survey Study - Patienten Perspectief (kinderen) - Vermoeidheid

Number	Question	Answers
	Geef een reactie op elke vraag of uitspraak door per rij één bolletje aan te klikken.	
	Geef alsjeblieft antwoord voor de afgelopen 7 dagen…	
2.1	Ik kon moeilijk bijblijven met mijn werk voor school omdat ik moe was	◯ Nooit ◯ Bijna nooit ◯ Soms ◯ Vaak ◯ Bijna altijd
2.2	Door mijn moeheid was het moeilijk om zo veel als ik zou willen te spelen of weg te gaan met mijn vrienden of vriendinnen .	◯ Nooit ◯ Bijna nooit ◯ Soms ◯ Vaak ◯ Bijna altijd
2.3	Ik voelde me zwak	◯ Nooit ◯ Bijna nooit ◯ Soms ◯ Vaak ◯ Bijna altijd
2.4	Ik werd gauw moe	◯ Nooit ◯ Bijna nooit ◯ Soms ◯ Vaak ◯ Bijna altijd
2.5	Ik had moeite om dingen af te maken omdat ik te moe was	◯ Nooit ◯ Bijna nooit ◯ Soms ◯ Vaak ◯ Bijna altijd
2.6	Ik was zo moe dat ik moeite had om op te letten	◯ Nooit ◯ Bijna nooit ◯ Soms ◯ Vaak ◯ Bijna altijd
2.7	Ik was te moe om te sporten of te bewegen	◯ Nooit ◯ Bijna nooit ◯ Soms ◯ Vaak ◯ Bijna altijd
2.8	Ik was te moe om buiten dingen te doen	◯ Nooit ◯ Bijna nooit ◯ Soms ◯ Vaak ◯ Bijna altijd
2.9	Ik was te moe om te genieten van de dingen die ik leuk vind om te doen	◯ Nooit ◯ Bijna nooit ◯ Soms ◯ Vaak ◯ Bijna altijd

Gitelman Survey Study - Patienten Perspectief (kinderen) - Angst

Number	Question	Answers
	Geef een reactie op elke vraag of uitspraak door per rij één bolletje aan te klikken.	
	Geef alsjeblieft antwoord voor de afgelopen 7 dagen.	
3.1	Ik had het gevoel dat er iets verschrikkelijks zou kunnen gebeuren	○ Nooit ○ Bijna nooit ○ Soms ○ Vaak ○ Bijna altijd
3.2	Ik voelde me zenuwachtig	○ Nooit ○ Bijna nooit ○ Soms ○ Vaak ○ Bijna altijd
3.3	Ik maakte me zorgen	○ Nooit ○ Bijna nooit ○ Soms ○ Vaak ○ Bijna altijd
3.4	Ik maakte me zorgen als ik thuis was	○ Nooit ○ Bijna nooit ○ Soms ○ Vaak ○ Bijna altijd
3.5	Ik voelde me bang	○ Nooit ○ Bijna nooit ○ Soms ○ Vaak ○ Bijna altijd
3.6	Ik maakte me zorgen als ik ’s avonds naar bed ging	○ Nooit ○ Bijna nooit ○ Soms ○ Vaak ○ Bijna altijd
3.7	Ik maakte me zorgen over wat er met me zou kunnen gebeuren	○ Nooit ○ Bijna nooit ○ Soms ○ Vaak ○ Bijna altijd
3.8	Ik werd echt gauw bang	○ Nooit ○ Bijna nooit ○ Soms ○ Vaak ○ Bijna altijd

Gitelman Survey Study - Patienten Perspectief (kinderen) - Cognitief functioneren

Number	Question	Answers
	Geef een reactie op elke vraag of uitspraak door per rij één bolletje aan te klikken.	
	Geef alsjeblieft antwoord voor de afgelopen 4 weken.	
4.1	Ik moet vaker dan leeftijdgenoten geschreven lijstje gebruiken, zodat ik geen dingen vergeet.	◯ Nooit ◯ Heel af en toe ◯ Af en toe ◯ Meestal ◯ Altijd
4.2	Het is moeilijk voor mij om me langer dan 5-10 minuten op één ding te concentreren.	◯ Nooit ◯ Heel af en toe ◯ Af en toe ◯ Meestal ◯ Altijd
4.3	Ik heb moeite om te onthouden wat ik aan het doen ben als ik onderbroken word.	◯ Nooit ◯ Heel af en toe ◯ Af en toe ◯ Meestal ◯ Altijd
4.4	Ik moet dingen een paar keer lezen voordat ik ze begrijp.	◯ Nooit ◯ Heel af en toe ◯ Af en toe ◯ Meestal ◯ Altijd
4.5	Ik vergeet gauw dingen.	◯ Nooit ◯ Heel af en toe ◯ Af en toe ◯ Meestal ◯ Altijd
4.6	Ik moet echt veel moeite doen om op te letten, anders maak ik fouten.	◯ Nooit ◯ Heel af en toe ◯ Af en toe ◯ Meestal ◯ Altijd
4.7	Ik heb moeite om me te herinneren dat ik dingen zoals projecten voor school of huishoudelijke klusjes moet doen.	◯ Nooit ◯ Heel af en toe ◯ Af en toe ◯ Meestal ◯ Altijd

Gitelman Survey Study - Patienten Perspectief (kinderen) - Lichamelijk functioneren

Number	Question	Answers
	Geef een reactie op elke vraag of uitspraak door per rij één bolletje aan te klikken.	
	Geef alsjeblieft antwoord voor de afgelopen 7 dagen.	
5.1	Ik kon van de vloer opstaan	◯ Zonder moeite ◯ Met een beetje moeite ◯ Met enige moeite ◯ Met veel moeite ◯ Kon het niet
5.2	Als ik met andere kinderen speelde kon ik ze bijhouden	◯ Zonder moeite ◯ Met een beetje moeite ◯ Met enige moeite ◯ Met veel moeite ◯ Kon het niet
5.3	Ik kon mijn benen bewegen	◯ Zonder moeite ◯ Met een beetje moeite ◯ Met enige moeite ◯ Met veel moeite ◯ Kon het niet
5.4	Ik kon zelf gaan staan	◯ Zonder moeite ◯ Met een beetje moeite ◯ Met enige moeite ◯ Met veel moeite ◯ Kon het niet
5.5	Ik kon op mijn tenen staan	◯ Zonder moeite ◯ Met een beetje moeite ◯ Met enige moeite ◯ Met veel moeite ◯ Kon het niet
5.6	Ik kon trappen opgaan zonder me ergens aan vast te houden	◯ Zonder moeite ◯ Met een beetje moeite ◯ Met enige moeite ◯ Met veel moeite ◯ Kon het niet
5.7	Ik was lichamelijk in staat de dingen te doen die ik het liefste doe	◯ Zonder moeite ◯ Met een beetje moeite ◯ Met enige moeite ◯ Met veel moeite ◯ Kon het niet

Gitelman Survey Study - Patienten Perspectief (kinderen) - Vermogen om een aandeel te hebben in sociale rollen en activiteiten

Number	Question	Answers
	Geef een reactie op elke vraag of uitspraak door per rij één bolletje aan te klikken.	
	Geef alsjeblieft antwoord voor de afgelopen 7 dagen.	
6.1	Ik voelde me geaccepteerd door andere kinderen van mijn leeftijd	○ Nooit ○ Bijna nooit ○ Soms ○ Vaak ○ Bijna altijd
6.2	Ik kon op mijn vrienden en vriendinnen rekenen.	○ Nooit ○ Bijna nooit ○ Soms ○ Vaak ○ Bijna altijd
6.3	Ik kon over alles praten met mijn vrienden of vriendinnen.	○ Nooit ○ Bijna nooit ○ Soms ○ Vaak ○ Bijna altijd
6.4	Ik kon makkelijk vrienden of vriendinnen maken.	○ Nooit ○ Bijna nooit ○ Soms ○ Vaak ○ Bijna altijd
6.5	Mijn vrienden en vriendinnen en ik hielpen elkaar.	○ Nooit ○ Bijna nooit ○ Soms ○ Vaak ○ Bijna altijd
6.6	Andere kinderen wilden mijn vriend of vriendin zijn.	○ Nooit ○ Bijna nooit ○ Soms ○ Vaak ○ Bijna altijd
6.7	Andere kinderen wilden bij me zijn.	○ Nooit ○ Bijna nooit ○ Soms ○ Vaak ○ Bijna altijd
6.8	Andere kinderen wilden met me praten.	○ Nooit ○ Bijna nooit ○ Soms ○ Vaak ○ Bijna altijd

Gitelman Survey Study - Patienten Perspectief (kinderen) - Slaapstoornissen

Number	Question	Answers
	Geef een reactie op elke vraag of uitspraak door per rij één bolletje aan te klikken.	
	Geef alsjeblieft antwoord voor de afgelopen 7 dagen.	
7.1	Ik had moeite met in slaap vallen	○ Nooit ○ Bijna nooit ○ Soms ○ Bijna altijd ○ Altijd
7.2	Ik sliep de hele nacht door	○ Nooit ○ Bijna nooit ○ Soms ○ Bijna altijd ○ Altijd
7.3	Ik had een slaapprobleem	○ Nooit ○ Bijna nooit ○ Soms ○ Bijna altijd ○ Altijd
7.4	Ik had moeite met slapen	○ Nooit ○ Bijna nooit ○ Soms ○ Bijna altijd ○ Altijd
7.5	Het duurde lang voordat ik in slaap viel.	○ Nooit ○ Bijna nooit ○ Soms ○ Bijna altijd ○ Altijd
7.6	Ik maakte met zorgen dat ik niet in slaap kon vallen	○ Nooit ○ Bijna nooit ○ Soms ○ Bijna altijd ○ Altijd
7.7	Ik werd ’s nachts wakker en had moeite om weer in slaap te vallen.	○ Nooit ○ Bijna nooit ○ Soms ○ Bijna altijd ○ Altijd
7.8	Ik lag ’s nachts te woelen	○ Nooit ○ Bijna nooit ○ Soms ○ Bijna altijd ○ Altijd

Gitelman Survey Study - Patienten Perspectief (kinderen) - Pijnintensiteit

Number	Question	Answers
	Geef alsjeblieft antwoord voor de afgelopen 7 dagen.	
8.1	Hoe erg was je pijn gemiddeld?	Geen pijn (0) ——— Ergste pijn die je kan bedenken (10)

Gitelman Survey Study - Patienten Perspectief (kinderen) - Gitelman specifieke klachten

Number	Question	Answers
	Beantwoord alsjeblieft elke vraag door één bolletje aan te duiden door het aan te klikken.	
	Geef per klacht aan hoeveel last je hier van hebt, van de afgelopen 7 dagen.	
9.1	Duizeligheid	○ Helemaal niet ○ Een beetje ○ Enigzins ○ Behoorlijk ○ Heel erg
9.2	Flauwvallen	○ Helemaal niet ○ Een beetje ○ Enigzins ○ Behoorlijk ○ Heel erg
9.3	Spierzwakte	○ Helemaal niet ○ Een beetje ○ Enigzins ○ Behoorlijk ○ Heel erg
9.4	Kramp	○ Helemaal niet ○ Een beetje ○ Enigzins ○ Behoorlijk ○ Heel erg
9.5	Plotseling samentrekken van mijn spieren	○ Helemaal niet ○ Een beetje ○ Enigzins ○ Behoorlijk ○ Heel erg
9.6	Spierstijfheid of pijn	○ Helemaal niet ○ Een beetje ○ Enigzins ○ Behoorlijk ○ Heel erg
9.7	Pijn in mijn gewrichten	○ Helemaal niet ○ Een beetje ○ Enigzins ○ Behoorlijk ○ Heel erg
9.8	Nachtelijk plassen	○ Helemaal niet ○ Een beetje ○ Enigzins ○ Behoorlijk ○ Heel erg
9.9	Veel plassen	○ Helemaal niet ○ Een beetje ○ Enigzins ○ Behoorlijk ○ Heel erg
9.10	Veel drinken	○ Helemaal niet ○ Een beetje ○ Enigzins ○ Behoorlijk ○ Heel erg
9.11	Ik voeg veel zout toe aan mijn eten	○ Helemaal niet ○ Een beetje ○ Enigzins ○ Behoorlijk ○ Heel erg
9.12	Overgeven	○ Helemaal niet ○ Een beetje ○ Enigzins ○ Behoorlijk ○ Heel erg

9.13	Ik kan moeilijk stoelgang maken	◯ Helemaal niet ◯ Een beetje ◯ Enigzins ◯ Behoorlijk ◯ Heel erg
9.14	Buikpijn	◯ Helemaal niet ◯ Een beetje ◯ Enigzins ◯ Behoorlijk ◯ Heel erg
9.15	Tintelingen	◯ Helemaal niet ◯ Een beetje ◯ Enigzins ◯ Behoorlijk ◯ Heel erg
9.16	Mijn hart slaat soms een slag over	◯ Helemaal niet ◯ Een beetje ◯ Enigzins ◯ Behoorlijk ◯ Heel erg

Gitelman Survey Study - Patienten Perspectief (kinderen) - Outro

Dankjewel!

Je hebt vragenlijst afgerond en daarbij bijgedragen aan onderzoek over Gitelman Syndroom.

Wij gaan verder aan de slag met de analyse, zo hopen we uiteindelijk een betere blik te krijgen over hoe Gitelman syndroom een impact heeft op jullie leven.

Groeten Het onderzoeksteam van de Gitelman Survey Studie

Survey 'Gitelman Survey Study - Patienten Perspectief (ouders)'

Gitelman Survey Study - Patienten Perspectief (ouders) - Introduction

Beste deelnemer

Dankjewel om deze vragenlijst in te vullen voor je kind! Het doel van onze studie is om een beter zicht te krijgen in hoe patienten met Gitelman syndroom hun ziekte ervaren. Dit doen we door een aantal stellingen over Gitelman syndroom (bijvoorbeeld de klachten) te bevragen in hoeverre jij je daarin kan vinden.

Het zou maximaal 30 minuten mogen duren om deze vragenlijst in te vullen. Je moet steeds een bolletje aanduiden wat jouw gevoel is ten aanzien van de stelling.

Alvast bedankt voor je bijdrage!

Groeten Het onderzoeksteam van de Gitelman Survey Studie

Gitelman Survey Study - Patienten Perspectief (ouders) - Beschrijving

Number	Question	Answers
1.1	Wat is het geslacht van uw kind?	○ Man<br>○ Vrouw
1.2	Wat is de leeftijd van uw kind?	[ ] jaar

Gitelman Survey Study - Patienten Perspectief (ouders) - Vermoeidheid

Number	Question	Answers
	Geef een reactie op elke vraag of uitspraak door per rij één bolletje aan te klikken.	
	Geef alstublieft antwoord voor de afgelopen 7 dagen.	
2.1	Door moeheid was het voor mijn kind moeilijk om zo veel als hij/zij zou willen te spelen of weg te gaan met zijn/haar vrienden of vriendinnen	◯ Nooit ◯ Bijna nooit ◯ Soms ◯ Vaak ◯ Bijna altlijd
2.2	Mijn kind voelde zich zwak.	◯ Nooit ◯ Bijna nooit ◯ Soms ◯ Vaak ◯ Bijna altijd
2.3	Mijn kind werd gauw moe	◯ Nooit ◯ Bijna nooit ◯ Soms ◯ Vaak ◯ Bijna altijd
2.4	Mijn kind kon moeilijk bijblijven met zijn/haar werk voor school omdat hij/zij moe was	◯ Nooit ◯ Bijna nooit ◯ Soms ◯ Vaak ◯ Bijna altijd
2.5	Mijn kind had moeite om dingen af te maken omdat hij/zij te moe was	◯ Nooit ◯ Bijna nooit ◯ Soms ◯ Vaak ◯ Bijna altijd
2.6	Mijn kind was zo moe dat hij/zij moeite had om op te letten	◯ Nooit ◯ Bijna nooit ◯ Soms ◯ Vaak ◯ Bijna altijd
2.7	Mijn kind was te moe om te sporten of te bewegen	◯ Nooit ◯ Bijna nooit ◯ Soms ◯ Vaak ◯ Bijna altijd
2.8	Mijn kind was te moe om buiten dingen te doen	◯ Nooit ◯ Bijna nooit ◯ Soms ◯ Vaak ◯ Bijna altijd
2.9	Mijn kind was te moe om te genieten van de dingen die hij/zij leuk vindt om te doen	◯ Nooit ◯ Bijna nooit ◯ Soms ◯ Vaak ◯ Bijna altlijd

Gitelman Survey Study - Patienten Perspectief (ouders) - Angst

Number	Question	Answers
	Geef een reactie op elke vraag of uitspraak door per rij één bolletje aan te klikken.	
	Geef alstublieft antwoord voor de afgelopen 7 dagen.	
3.1	Mijn kind voelde zich zenuwachtig	◯ Nooit ◯ Bijna nooit ◯ Soms ◯ Vaak ◯ Bijna altijd
3.2	Mijn kind voelde zich bang	◯ Nooit ◯ Bijna nooit ◯ Soms ◯ Vaak ◯ Bijna altijd
3.3	Mijn kind maakte zich zorgen	◯ Nooit ◯ Bijna nooit ◯ Soms ◯ Vaak ◯ Bijna altijd
3.4	Mijn kind had het gevoel dat er iets verschrikkelijks zou kunnen gebeuren	◯ Nooit ◯ Bijna nooit ◯ Soms ◯ Vaak ◯ Bijna altijd
3.5	Mijn kind maakte zich zorgen als hij/zij thuis was	◯ Nooit ◯ Bijna nooit ◯ Soms ◯ Vaak ◯ Bijna altijd
3.6	Mijn kind werd echt gauw bang	◯ Nooit ◯ Bijna nooit ◯ Soms ◯ Vaak ◯ Bijna altijd
3.7	Mijn kind maakte zich zorgen over wat er met hem/haar zou kunnen gebeuren	◯ Nooit ◯ Bijna nooit ◯ Soms ◯ Vaak ◯ Bijna altijd
3.8	Mijn kind maakte zich zorgen als hij/zij ’s avonds naar bed ging	◯ Nooit ◯ Bijna nooit ◯ Soms ◯ Vaak ◯ Bijna altijd

Gitelman Survey Study - Patienten Perspectief (ouders) - Cognitief functioneren

Number	Question	Answers
	Geef een reactie op elke vraag of uitspraak door per rij één bolletje aan te klikken.	
	Geef alstublieft antwoord voor de afgelopen 4 weken	
4.1	Uw kind moet vaker dan leeftijdgenoten geschreven lijstje gebruiken, zodat hij/zij geen dingen vergeet.	◯ Nooit ◯ Heel af en toe ◯ Af en toe ◯ Meestal ◯ Altijd
4.2	Het is moeilijk voor uw kind om zich langer dan 5-10 minuten op één ding te concentreren.	◯ Nooit ◯ Heel af en toe ◯ Af en toe ◯ Meestal ◯ Altijd
4.3	Uw kind heeft moeite om te onthouden wat hij/zij aan het doen is als hij/zij onderbroken wordt.	◯ Nooit ◯ Heel af en toe ◯ Af en toe ◯ Meestal ◯ Altijd
4.4	Uw kind moet dingen een paar keer lezen voordat hij/zij ze begrijpt.	◯ Nooit ◯ Heel af en toe ◯ Af en toe ◯ Meestal ◯ Altijd
4.5	Uw kind vergeet gauw dingen.	◯ Nooit ◯ Heel af en toe ◯ Af en toe ◯ Meestal ◯ Altijd
4.6	Uw kind moet echt veel moeite doen om op te letten, anders maakt hij/zij fouten.	◯ Nooit ◯ Heel af en toe ◯ Af en toe ◯ Meestal ◯ Altijd
4.7	Uw kind heeft moeite om zich te herinneren dat hij/zij dingen zoals projecten voor school of huishoudelijke klusjes moet doen.	◯ Nooit ◯ Heel af en toe ◯ Af en toe ◯ Meestal ◯ Altijd

Gitelman Survey Study - Patienten Perspectief (ouders) - Lichamelijk functioneren

Number	Question	Answers
	Beantwoord alstublieft elke vraag door één bolletje aan te duiden door het aan te klikken.	
	Geef a.u.b. antwoord voor de afgelopen 7 dagen.	
5.1	Mijn kind kon van de vloer opstaan	◯ Zonder moeite ◯ Met een beetje moeite ◯ Met enige moeite ◯ Met veel moeite ◯ Kon het niet
5.2	Als mijn kind met andere kinderen speelde, kon hij/zij ze bijhouden	◯ Zonder moeite ◯ Met een beetje moeite ◯ Met enige moeite ◯ Met veel moeite ◯ Kon het niet
5.3	Mijn kind kon zijn/haar benen bewegen	◯ Zonder moeite ◯ Met een beetje moeite ◯ Met enige moeite ◯ Met veel moeite ◯ Kon het niet
5.4	Mijn kind kon zelf gaan staan	◯ Zonder moeite ◯ Met een beetje moeite ◯ Met enige moeite ◯ Met veel moeite ◯ Kon het niet
5.5	Mijn kind kon op zijn/haar tenen staan	◯ Zonder moeite ◯ Met een beetje moeite ◯ Met enige moeite ◯ Met veel moeite ◯ Kon het niet
5.6	Mijn kind kon trappen opgaan zonder zich ergens aan vast te houden	◯ Zonder moeite ◯ Met een beetje moeite ◯ Met enige moeite ◯ Met veel moeite ◯ Kon het niet
5.7	Mijn kind was lichamelijk in staat de dingen te doen die hij/zij het liefste doet	◯ Zonder moeite ◯ Met een beetje moeite ◯ Met enige moeite ◯ Met veel moeite ◯ Kon het niet

Gitelman Survey Study - Patienten Perspectief (ouders) - Vermogen om een aandeel te hebben in sociale rollen en activiteiten

Number	Question	Answers
	Geef een reactie op elke vraag of uitspraak door per rij één bolletje aan te klikken.	
	Geef alstublieft antwoord voor de afgelopen 7 dagen	
6.1	Mijn kind voelde zich geaccepteerd door andere kinderen van zijn/haar leeftijd	◯ Nooit ◯ Bijna nooit ◯ Soms ◯ Vaak ◯ Bijna altijd
6.2	Mijn kind kon op zijn/haar vrienden en vriendinnen rekenen	◯ Nooit ◯ Bijna nooit ◯ Soms ◯ Vaak ◯ Bijna altijd
6.3	Mijn kind kon makkelijk vrienden of vriendinnen maken	◯ Nooit ◯ Bijna nooit ◯ Soms ◯ Vaak ◯ Bijna altijd
6.4	Mijn kind en zijn/haar vrienden en vriendinnen hielpen elkaar	◯ Nooit ◯ Bijna nooit ◯ Soms ◯ Vaak ◯ Bijna altijd
6.5	Andere kinderen wilden de vriend of vriendin van mij kind zijn	◯ Nooit ◯ Bijna nooit ◯ Soms ◯ Vaak ◯ Bijna altijd
6.6	Andere kinderen wilden bij mijn kind zijn	◯ Nooit ◯ Bijna nooit ◯ Soms ◯ Vaak ◯ Bijna altijd
6.7	Andere kinderen wilden met mijn kind praten.	◯ Nooit ◯ Bijna nooit ◯ Soms ◯ Vaak ◯ Bijna altijd

Gitelman Survey Study - Patienten Perspectief (ouders) - Slaapstoornissen

Number	Question	Answers
	Geef een reactie op elke vraag of uitspraak door per rij één bolletje aan te klikken.	
	Geef alstublieft antwoord voor de afgelopen 7 dagen	
7.1	Mijn kind had moeite met in slaap vallen	◯ Nooit ◯ Bijna nooit ◯ Soms ◯ Bijna altijd ◯ Altijd
7.2	Mijn kind sliep de hele nacht door	◯ Nooit ◯ Zelden ◯ Soms ◯ Meestal ◯ Altijd
7.3	Mijn kind had een slaapprobleem	◯ Nooit ◯ Bijna nooit ◯ Soms ◯ Bijna altijd ◯ Altijd
7.4	Mijn kind had moeite met slapen	◯ Nooit ◯ Bijna nooit ◯ Soms ◯ Bijna altijd ◯ Altijd
7.5	Het duurde lang voordat mijn kind in slaap viel	◯ Nooit ◯ Bijna nooit ◯ Soms ◯ Bijna altijd ◯ Altijd
7.6	Mijn kind maakte zich zorgen dat hij/zij niet in slaap kon vallen	◯ Nooit ◯ Bijna nooit ◯ Soms ◯ Bijna altijd ◯ Altijd
7.7	Mijn kind werd ’s nachts wakker en had moeite om weer in slaap te vallen	◯ Nooit ◯ Bijna nooit ◯ Soms ◯ Bijna altijd ◯ Altijd
7.8	Mijn kind lag ’s nachts te woelen	◯ Nooit ◯ Bijna nooit ◯ Soms ◯ Bijna altijd ◯ Altijd

Gitelman Survey Study - Patienten Perspectief (ouders) - Pijnintensiteit

Number	Question	Answers
	Geef a.u.b. antwoord voor de afgelopen 7 dagen.	
8.1	Hoe erg was de pijn van uw kind gemiddeld?	Geen pijn (0) — Ergst pijn die u kan bedenken (10)

Gitelman Survey Study - Patienten Perspectief (ouders) - Gitelman specifieke klachten

Number	Question	Answers
	Beantwoord alstublieft elke vraag door één bolletje aan te duiden door het aan te klikken.	
	Geef a.u.b. per symptoom aan hoeveel last uw kind hier van had, van de afgelopen 7 dagen.	
9.1	Duizeligheid	◯ Helemaal niet ◯ Een beetje ◯ Enigzins ◯ Behoorlijk ◯ Heel erg
9.2	Flauwvallen	◯ Helemaal niet ◯ Een beetje ◯ Enigzins ◯ Behoorlijk ◯ Heel erg
9.3	Spierzwakte	◯ Helemaal niet ◯ Een beetje ◯ Enigzins ◯ Behoorlijk ◯ Heel erg
9.4	Kramp	◯ Helemaal niet ◯ Een beetje ◯ Enigzins ◯ Behoorlijk ◯ Heel erg
9.5	Spierspasme	◯ Helemaal niet ◯ Een beetje ◯ Enigzins ◯ Behoorlijk ◯ Heel erg
9.6	Spierstijfheid of pijn	◯ Helemaal niet ◯ Een beetje ◯ Enigzins ◯ Behoorlijk ◯ Heel erg
9.7	Gewrichtspijn	◯ Helemaal niet ◯ Een beetje ◯ Enigzins ◯ Behoorlijk ◯ Heel erg
9.8	Nachtelijk plassen	◯ Helemaal niet ◯ Een beetje ◯ Enigzins ◯ Behoorlijk ◯ Heel erg
9.9	Veel plassen	◯ Helemaal niet ◯ Een beetje ◯ Enigzins ◯ Behoorlijk ◯ Heel erg
9.10	Veel drinken	◯ Helemaal niet ◯ Een beetje ◯ Enigzins ◯ Behoorlijk ◯ Heel erg
9.11	Zouthonger	◯ Helemaal niet ◯ Een beetje ◯ Enigzins ◯ Behoorlijk ◯ Heel erg
9.12	Braken	◯ Helemaal niet ◯ Een beetje ◯ Enigzins ◯ Behoorlijk ◯ Heel erg

9.13	Constipatie	◯ Helemaal niet ◯ Een beetje ◯ Enigzins ◯ Behoorlijk ◯ Heel erg
9.14	Buikpijn	◯ Helemaal niet ◯ Een beetje ◯ Enigzins ◯ Behoorlijk ◯ Heel erg
9.15	Tintelingen	◯ Helemaal niet ◯ Een beetje ◯ Enigzins ◯ Behoorlijk ◯ Heel erg
9.16	Hartkloppingen	◯ Helemaal niet ◯ Een beetje ◯ Enigzins ◯ Behoorlijk ◯ Heel erg

Gitelman Survey Study - Patienten Perspectief (ouders) - Outro

Dankjewel!

Je hebt vragenlijst afgerond en daarbij bijgedragen aan onderzoek over Gitelman Syndroom.

Wij gaan verder aan de slag met de analyse, zo hopen we uiteindelijk een betere blik te krijgen over hoe Gitelman syndroom een impact heeft op jullie leven.

Groeten Het onderzoeksteam van de Gitelman Survey Studie

Survey 'Gitelman Survey Study - Patienten Perspectief (volwassenen)'

Gitelman Survey Study - Patienten Perspectief (volwassenen) - Introduction

Beste deelnemer

Dankjewel om deze vragenlijst in te vullen! Het doel van onze studie is om een beter zicht te krijgen in hoe patienten met Gitelman syndroom hun ziekte ervaren. Dit doen we door een aantal stellingen over Gitelman syndroom (bijvoorbeeld de klachten) te bevragen in hoeverre jij je daarin kan vinden.

Het zou maximaal 30 minuten mogen duren om deze vragenlijst in te vullen. Je moet steeds een bolletje aanduiden wat jouw gevoel is ten aanzien van de stelling.

Alvast bedankt voor je bijdrage!

Groeten Het onderzoeksteam van de Gitelman Survey Studie

Gitelman Survey Study - Patienten Perspectief (volwassenen) - Beschrijving

Number	Question	Answers
1.1	Wat is uw geslacht?	◯ Man<br>◯ Vrouw
1.2	Wat is uw leeftijd?	[ ] Jaar

Gitelman Survey Study - Patienten Perspectief (volwassenen) - Vermoeidheid

Number	Question	Answers
	Beantwoord alstublieft elke vraag door één bolletje aan te duiden door het aan te klikken.	
	Geef a.u.b. antwoord voor de afgelopen 7 dagen.	
2.1	Ik heb last van vermoeidheid	◯ Helemaal niet ◯ Een beetje ◯ Enigzins ◯ In vrij hoge mate ◯ In zeer hoge mate
2.2	Het kost me moeite om met dingen te **beginnen** omdat ik zo moe ben.	◯ Helemaal niet ◯ Een beetje ◯ Enigzins ◯ In vrij hoge mate ◯ In zeer hoge mate
2.3	Hoe afgepeigerd voelde u zich gemiddeld genomen?	◯ Helemaal niet ◯ Een beetje ◯ Enigzins ◯ Behoorlijk ◯ Heel erg
2.4	Hoe vermoeid was u gemiddeld genomen?	◯ Helemaal niet ◯ Een beetje ◯ Enigzins ◯ Behoorlijk ◯ Heel erg
2.5	Hoezeer had u gemiddeld genomen last van uw vermoeidheid?	◯ Helemaal niet ◯ Een beetje ◯ Enigzins ◯ Behoorlijk ◯ Heel erg
2.6	In welke mate belemmerde uw vermoeidheid u bij het lichamelijk functioneren?	◯ Helemaal niet ◯ Een beetje ◯ Enigzins ◯ Behoorlijk ◯ Heel erg
2.7	Hoe vaak was het, vanwege uw vermoeidheid, nodig uzelf aan te sporen om dingen af te krijgen?	◯ Nooit ◯ Zelden ◯ Soms ◯ Vaak ◯ Altijd
2.8	Hoe vaak had u vanwege uw vermoeidheid moeite om dingen af te krijgen?	◯ Nooit ◯ Zelden ◯ Soms ◯ Vaak ◯ Altijd

Gitelman Survey Study - Patienten Perspectief (volwassenen) - Angst

Number	Question	Answers
	Beantwoord alstublieft elke vraag door één bolletje aan te duiden door het aan te klikken.	
	Geef a.u.b. antwoord voor de afgelopen 7 dagen.	
3.1	Ik voelde me angstig.	◯ Nooit ◯ Zelden ◯ Soms ◯ Vaak ◯ Altijd
3.2	Ik vond het moeilijk om me op iets anders dan mijn angst en bezorgdheid te concentreren.	◯ Nooit ◯ Zelden ◯ Soms ◯ Vaak ◯ Altijd
3.3	Mijn zorgen waren me te veel.	◯ Nooit ◯ Zelden ◯ Soms ◯ Vaak ◯ Altijd
3.4	Ik voelde me slecht op mijn gemak.	◯ Nooit ◯ Zelden ◯ Soms ◯ Vaak ◯ Altijd
3.5	Ik voelde me zenuwachtig.	◯ Nooit ◯ Zelden ◯ Soms ◯ Vaak ◯ Altijd
3.6	Ik had het gevoel dat ik hulp nodig had voor mijn angst en bezorgdheid.	◯ Nooit ◯ Zelden ◯ Soms ◯ Vaak ◯ Altijd
3.7	Ik voelde me angstig en bezorgd.	◯ Nooit ◯ Zelden ◯ Soms ◯ Vaak ◯ Altijd
3.8	Ik voelde me gespannen.	◯ Nooit ◯ Zelden ◯ Soms ◯ Vaak ◯ Altijd

Gitelman Survey Study - Patienten Perspectief (volwassenen) - Cognitief functioneren

Number	Question	Answers
	Beantwoord alstublieft elke vraag door één bolletje aan te duiden door het aan te klikken.	
	Geef a.u.b. antwoord voor de afgelopen 7 dagen.	
4.1	Mijn denkproces verliep traag.	◯ Nooit ◯ Zelden (één keer) ◯ Soms (twee tot drie keer) ◯ Vaak (ongeveer één keer per dag) ◯ Zeer vaak (meerdere keren per dag)
4.2	Het leek alsof mijn hersenen niet zo goed werkten als gewoonlijk.	◯ Nooit ◯ Zelden (één keer) ◯ Soms (twee tot drie keer) ◯ Vaak (ongeveer één keer per dag) ◯ Zeer vaak (meerdere keren per dag)
4.3	Ik heb me méér moeten inspannen dan gewoonlijk om bij te houden wat ik aan het doen was.	◯ Nooit ◯ Zelden (één keer) ◯ Soms (twee tot drie keer) ◯ Vaak (ongeveer één keer per dag) ◯ Zeer vaak (meerdere keren per dag)
4.4	Ik had moeite om heen en weer te schakelen tussen verschillende activiteiten waarbij ik moest nadenken.	◯ Nooit ◯ Zelden (één keer) ◯ Soms (twee tot drie keer) ◯ Vaak (ongeveer één keer per dag) ◯ Zeer vaak (meerdere keren per dag)
4.5	Ik had moeite om me te concentreren	◯ Nooit ◯ Zelden (één keer) ◯ Soms (twee tot drie keer) ◯ Vaak (ongeveer één keer per dag) ◯ Zeer vaak (meerdere keren per dag)
4.6	Ik moest me echt heel hard inspannen om op te letten, anders zou ik een fout maken.	◯ Nooit ◯ Zelden (één keer) ◯ Soms (twee tot drie keer) ◯ Vaak (ongeveer één keer per dag) ◯ Zeer vaak (meerdere keren per dag)
4.7	Ik had moeite om mijn gedachten te vormen	◯ Nooit ◯ Zelden (één keer) ◯ Soms (twee tot drie keer) ◯ Vaak (ongeveer één keer per dag) ◯ Zeer vaak (meerdere keren per dag)

4.8	Ik had moeite met het optellen en aftrekken van getallen in mijn hoofd	◯ Nooit ◯ Zelden (één keer) ◯ Soms (twee tot drie keer) ◯ Vaak (ongeveer één keer per dag) ◯ Zeer vaak (meerdere keren per dag)

Gitelman Survey Study - Patienten Perspectief (volwassenen) - Lichamelijk functioneren

Number	Question	Answers
	Beantwoord alstublieft elke vraag door één bolletje aan te duiden door het aan te klikken.	
	Geef a.u.b. antwoord voor de afgelopen 7 dagen.	
5.1	Kunt u klusjes doen zoals stofzuigen of in de tuin werken?	◯ Zonder moeite ◯ Met een beetje moeite ◯ Met enige moeite ◯ Met veel moeite ◯ Kan het niet
5.2	Kunt u in een normaal tempo trappen open afgaan?	◯ Zonder moeite ◯ Met een beetje moeite ◯ Met enige moeite ◯ Met veel moeite ◯ Kan het niet
5.3	Kunt u een wandeling van ten minste 15 minuten maken?	◯ Zonder moeite ◯ Met een beetje moeite ◯ Met enige moeite ◯ Met veel moeite ◯ Kan het niet
5.4	Kunt u boodschappen doen en winkelen?	◯ Zonder moeite ◯ Met een beetje moeite ◯ Met enige moeite ◯ Met veel moeite ◯ Kan het niet
5.5	Wordt u door uw gezondheid op dit moment beperkt in het verrichten van twee uur lichamelijke arbeid?	◯ Helemaal niet ◯ Heel weinig ◯ Enigszins ◯ Behoorlijk ◯ Kan het niet
5.6	Wordt u door uw gezondheid op dit moment beperkt in het uitvoeren van matig zwaar werk in en om het huis, zoals stofzuigen, vloeren vegen of boodschappen naar binnen dragen?	◯ Helemaal niet ◯ Heel weinig ◯ Enigszins ◯ Behoorlijk ◯ Kan het niet
5.7	Wordt u door uw gezondheid op dit moment beperkt in het tillen of dragen van boodschappen?	◯ Helemaal niet ◯ Heel weinig ◯ Enigszins ◯ Behoorlijk ◯ Kan het niet
5.8	Wordt u door uw gezondheid op dit moment beperkt in het uitvoeren van zwaar werk in en om het huis, zoals vloeren schrobben, of tillen of verplaatsen van zware meubels?	◯ Helemaal niet ◯ Heel weinig ◯ Enigszins ◯ Behoorlijk ◯ Kan het niet

Gitelman Survey Study - Patienten Perspectief (volwassenen) - Vermogen om een aandeel te hebben in sociale rollen en activiteiten

Number	Question	Answers
	Beantwoord alstublieft elke vraag door één bolletje aan te duiden door het aan te klikken.	
	Geef a.u.b. antwoord voor de afgelopen 7 dagen.	
6.1	Ik heb moeite om al mijn gewone vrijetijdsactiviteiten met anderen te doen .	○ Nooit ○ Zelden ○ Soms ○ Meestal ○ Altijd
6.2	Ik heb moeite om alle gezins- /familieactiviteiten te doen die ik wil doen.	○ Nooit ○ Zelden ○ Soms ○ Meestal ○ Altijd
6.3	Ik heb moeite om al mijn gewone werk (inclusief werk thuis) te doen.	○ Nooit ○ Zelden ○ Soms ○ Meestal ○ Altijd
6.4	Ik heb moeite om alle activiteiten met vrienden te doen die ik wil doen.	○ Nooit ○ Zelden ○ Soms ○ Meestal ○ Altijd
6.5	Ik moet de leuke dingen die ik met anderen doe beperken.	○ Nooit ○ Zelden ○ Soms ○ Meestal ○ Altijd
6.6	Ik moet mijn gewone activiteiten met vrienden beperken.	○ Nooit ○ Zelden ○ Soms ○ Meestal ○ Altijd
6.7	Ik moet mijn gewone gezins- /familieactiviteiten beperken.	○ Nooit ○ Zelden ○ Soms ○ Meestal ○ Altijd
6.8	Ik heb moeite om al het werk (inclusief werk thuis) te doen dat echt belangrijk is voor mij.	○ Nooit ○ Zelden ○ Soms ○ Meestal ○ Altijd

Gitelman Survey Study - Patienten Perspectief (volwassenen) - Slaapstoornissen

Number	Question	Answers
	Beantwoord alstublieft elke vraag door één bolletje aan te duiden door het aan te klikken.	
	Geef a.u.b. antwoord voor de afgelopen 7 dagen.	
7.1	De kwaliteit van mijn slaap was…	◯ Heel slecht ◯ Slecht ◯ Redelijk ◯ Goed ◯ Heel goed
7.2	Mijn slaap was verkwikkend.	◯ Helemaal niet ◯ Een beetje ◯ Enigszins ◯ Behoorlijk ◯ Heel erg
7.3	Ik had een slaapprobleem.	◯ Helemaal niet ◯ Een beetje ◯ Enigzins ◯ Behoorlijk ◯ Heel erg
7.4	Ik had moeite met in slaap vallen.	◯ Helemaal niet ◯ Een beetje ◯ Enigzins ◯ Behoorlijk ◯ Heel erg
7.5	Mijn slaap was onrustig.	◯ Helemaal niet ◯ Een beetje ◯ Enigzins ◯ Behoorlijk ◯ Heel erg
7.6	Ik deed mijn uiterste best om in slaap te vallen.	◯ Helemaal niet ◯ Een beetje ◯ Enigzins ◯ Behoorlijk ◯ Heel erg
7.7	Ik maakte me zorgen dat ik niet in slaap kon vallen.	◯ Helemaal niet ◯ Een beetje ◯ Enigzins ◯ Behoorlijk ◯ Heel erg
7.8	Ik was tevreden met mijn slaap.	◯ Helemaal niet ◯ Een beetje ◯ Enigszins ◯ Behoorlijk ◯ Heel erg

Gitelman Survey Study - Patienten Perspectief (volwassenen) - Pijnintensiteit

Number	Question	Answers
	Geef a.u.b. antwoord voor de afgelopen 7 dagen.	
8.1	Hoe zou u gemiddeld uw pijn beoordelen?	Geen pijn (0) — Ergst denkbare pijn (10)

Gitelman Survey Study - Patienten Perspectief (volwassenen) - Gitelman specifieke klachten

Number	Question	Answers
	Beantwoord alstublieft elke vraag door één bolletje aan te duiden door het aan te klikken.	
	Geef a.u.b. per symptoom aan hoeveel last u hier van heeft, van de afgelopen 7 dagen.	
9.1	Duizeligheid	◯ Helemaal niet ◯ Een beetje ◯ Enigzins ◯ Behoorlijk ◯ Heel erg
9.2	Flauwvallen	◯ Helemaal niet ◯ Een beetje ◯ Enigzins ◯ Behoorlijk ◯ Heel erg
9.3	Spierzwakte	◯ Helemaal niet ◯ Een beetje ◯ Enigzins ◯ Behoorlijk ◯ Heel erg
9.4	Kramp	◯ Helemaal niet ◯ Een beetje ◯ Enigzins ◯ Behoorlijk ◯ Heel erg
9.5	Spierspasme	◯ Helemaal niet ◯ Een beetje ◯ Enigzins ◯ Behoorlijk ◯ Heel erg
9.6	Spierstijfheid of pijn	◯ Helemaal niet ◯ Een beetje ◯ Enigzins ◯ Behoorlijk ◯ Heel erg
9.7	Gewrichtspijn	◯ Helemaal niet ◯ Een beetje ◯ Enigzins ◯ Behoorlijk ◯ Heel erg
9.8	Nachtelijk plassen	◯ Helemaal niet ◯ Een beetje ◯ Enigzins ◯ Behoorlijk ◯ Heel erg
9.9	Veel plassen	◯ Helemaal niet ◯ Een beetje ◯ Enigzins ◯ Behoorlijk ◯ Heel erg
9.10	Veel drinken	◯ Helemaal niet ◯ Een beetje ◯ Enigzins ◯ Behoorlijk ◯ Heel erg
9.11	Zouthonger	◯ Helemaal niet ◯ Een beetje ◯ Enigzins ◯ Behoorlijk ◯ Heel erg
9.12	Braken	◯ Helemaal niet ◯ Een beetje ◯ Enigzins ◯ Behoorlijk ◯ Heel erg

9.13	Constipatie	◯ Helemaal niet ◯ Een beetje ◯ Enigzins ◯ Behoorlijk ◯ Heel erg
9.14	Buikpijn	◯ Helemaal niet ◯ Een beetje ◯ Enigzins ◯ Behoorlijk ◯ Heel erg
9.15	Tintelingen	◯ Helemaal niet ◯ Een beetje ◯ Enigzins ◯ Behoorlijk ◯ Heel erg
9.16	Hartkloppingen	◯ Helemaal niet ◯ Een beetje ◯ Enigzins ◯ Behoorlijk ◯ Heel erg

Gitelman Survey Study - Patienten Perspectief (volwassenen) - Outro

Dankjewel!

Je hebt vragenlijst afgerond en daarbij bijgedragen aan onderzoek over Gitelman Syndroom.

Wij gaan verder aan de slag met de analyse, zo hopen we uiteindelijk een betere blik te krijgen over hoe Gitelman syndroom een impact heeft op jullie leven.

Groeten Het onderzoeksteam van de Gitelman Survey Studie
